# Synthesis of 5-arylidenerhodanines in L-proline-based deep eutectic solvent

**DOI:** 10.3762/bjoc.19.110

**Published:** 2023-10-04

**Authors:** Stéphanie Hesse

**Affiliations:** 1 Université de Lorraine, LCP-A2MC, F-57000, Metz, France

**Keywords:** antioxidant, deep eutectic solvent, Knoevenagel, ʟ-proline DES, rhodanine

## Abstract

Rhodanines and their derivatives are known to have many pharmacological activities that can be modulated through different functionalization sites. One of the most studied modification in those scaffolds is the introduction of a benzylidene moiety on C5 via a Knoevenagel reaction. Here, a facile synthesis of 5-arylidenerhodanines via a Knoevenagel reaction in an ʟ-proline-based deep eutectic solvent (DES) is reported. This method is fast (1 h at 60 °C), easy, catalyst-free and sustainable as no classical organic solvents were used. The expected compounds are recovered by a simple filtration after hydrolysis and no purification is required. Those derivatives were studied for their antioxidant activities and the results are consistent with those reported in the literature indicating that phenolic compounds are the more active ones.

## Introduction

**Figure 1:**
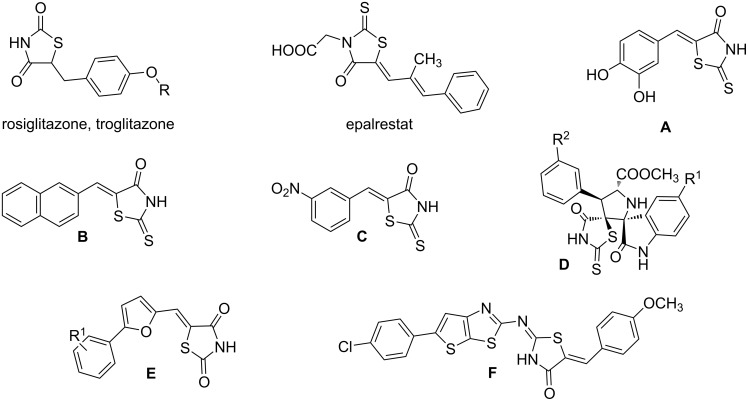
Examples of rhodanines and related five-membered heterocycles with interesting biological activities.

Rhodanines and related five-membered heterocycles with multiple heteroatoms (i.e., thiazolidinediones, thiazolidinones, hydantoins, thiohydantoins) are very interesting classes of heterocyclic compounds studied for a long time. Those scaffolds have attracted great attention since the introduction of various glitazones and epalrestat into clinical use for the treatment of type-II diabetes (Figure 1). Indeed, those compounds are often identified as hits in high throughput screenings and they present a wide spectrum of pharmacological activities [1–2]. Thus, for example, 3,4-dihydroxybenzylidenerhodanine (**A**) showed a high antioxidant activity with 71.2% of 1,1-diphenyl-2-picrylhydrazyl radical (DPPH) scavenging activity [3]. Naphthalen-2-ylmethylidenerhodanine (**B**) has been reported as inhibitor of chikungunya virus (IC_50_ of 3.6 µM) [4] whereas 3-nitrobenzylidenerhodanine (**C**) displayed a very potent antitubercular activity with a MIC of 0.05 µg/mL (compared to streptomycin MIC of 6.25 µg/mL) [5]. 5-Benzylidenerhodanine derivatives also constitute interesting starting compounds and allow, for example, the formation of rhodanine-fused spiro[pyrrolidine-2,3′-oxindoles] **D** having antidiabetic activity [6]. Thiazolidinediones and thiazolidinones were found to be potent moieties of a series of furan-2-ylmethylenethiazolidinediones **E** that were studied as selective ATP-competitive PI3Kγ inhibitors [7]. A few years ago, we worked intensively on 2-heteroarylimino-1,3-thiazolidin-4-ones as potential antitumor agents [8] and we demonstrated that derivative **F** was an interesting CDC25A inhibitor [9].

Up to now, the synthesis of most organic compounds still uses harmful reagents, volatile solvents, hard conditions, and/or difficult purifications. However, green chemistry has become a crucial sub-discipline in the field of chemistry and the chemical industry is giving major priority to sustainable processes. Since a few years, deep eutectic solvents (DES) are considered as a promising emerging class of green solvents as they offer numerous advantages, such as low volatility, non-flammability, chemical and thermal stability, recyclability, and above all a good biodegradability [10]. Moreover, their synthesis is usually easy and cheap as DES are formed by simply mixing an H-bond donor (HBD) and an H-bond acceptor (HBA) in appropriate molar ratios, generally at moderate temperature. A wide variety of DES is discussed in the literature depending on the individual components chosen. Several reviews on DES were published during the last years and clearly demonstrate the high potential of those solvents in many applications (electrodeposition, organic syntheses, biomass extraction, etc.) [11–12]. The more recent ones also deal with the importance of a better characterization of DES [13] and a clear evaluation of their sustainability via life-cycle assessment to evaluate the potential environmental impacts [14].

For a synthetic purpose, the melting point of the DES and its viscosity are key parameters for the convenience of their use. Indeed, DES viscosities are generally higher than those of water and other common organic solvents and a too high viscosity can act as an obstacle to their use as solvent in syntheses. Acidity and alkalinity of DES may also have a significant impact in designing organic reactions. In 2003, Abbott described a DES formed by combination of choline chloride (ChCl) and urea in a 1:2 ratio with a melting point of 12 °C [15]. This DES was further characterized; a pH value of 10.07 was measured at 30 °C by Shah et al. [16] and a viscosity of 750 cP at 25 °C was reported by Mjalli et al. [17] (Table 1). It remains today one of the most used DES and studies on it are still conducted [14]. ChCl/glycerol (ChCl/Gly; 1:2) is another classical DES with a melting point of 17 °C which is more acidic and less viscous than ChCl/urea [18–19] (Table 1). In addition, proline-based natural deep eutectic solvents (NaDES) were also studied and it was shown that they presented higher viscosity values than the ChCl-based NaDES, suggesting that the HBA used for the synthesis of NaDES plays a major role in the resulting viscosity [20]. For example, ʟ-proline/glycerol (Pro/Gly; 1:2) was found to have a pH value of 7.25 and a viscosity of 5064 cP [20].

**Table 1:** Comparison of physicochemical properties of selected DES used in this study.

DES	mp	pH	viscosity at 25 °C	reference

ChCl/urea (1:2)	12 °C	10.07 (at 30 °C)	750 cP	[15–17]
ChCl/Gly (1:2)	17 °C	4.47 (at 25 °C)	281 cP	[18–19]
Pro/Gly (1:2)	<20 °C^a^	7.25 (at 25 °C)	5064 cP	[20]

^a^Data not available – DES liquid at 20 °C.

In 2018, Molnar et al. reported the antioxidant activity of a series of rhodanine derivatives synthesized by a Knoevenagel condensation of rhodanine with different aldehydes [3]. The reactions were performed in ChCl/urea (1:2) at 90 °C, without needing a catalyst and the products were obtained in low to good yields (10–78%). On another hand, ʟ-proline is well known as an organocatalyst and its use in aldol and Knoevenagel condensation is well documented [21]. Moreover, the low cost and high availability of ʟ-proline has attracted attention to ʟ-proline-based DES. Especially, in 2022, Detsi [20] has synthesized and characterized three ʟ-proline-based NaDES: proline/oxalic acid (1:1), proline/glycerol (1:2), and proline/lactic acid/water (1:2:2.5). The authors studied their use in the synthesis of aurones via a Knoevenagel condensation and compared them to the classical choline-based DES, ChCl/Gly (1:2). They demonstrated that the ʟ-proline-based DES were superior to ChCl/Gly and obtained aurones from the reaction of benzofuranone and aldehydes in good to excellent yields in a few minutes under ultrasound irradiation.

## Results and Discussion

As a part of our ongoing research in DES chemistry, we were mainly interested in the conditions reported by Detsi [20], and attempted to apply them for rhodanine derivative synthesis. In accordance with the principles of green chemistry, we envisaged a procedure with temperatures not too high and reaction times not too long. We also wanted to isolate pure compounds by a simple filtration after precipitation in aqueous media without the need for extraction or purification steps as a real environmentally benign synthetic process should as far as possible exclude organic solvents from all stages. In a first attempt, vanillin was reacted with rhodanine for 2 h at room temperature in Pro/Gly (1:2) and product **3a** was obtained in 57% yield by a simple filtration after hydrolysis (Table 2, entry 1). The same compound was synthesized by Molnar et al. in 53% yield after reaction in ChCl/urea at 90 °C [3]. When increasing the reaction temperature to 60 °C, product **3a** was obtained with 94% yield after 1 h of reaction in Pro/Gly (1:2) (Table 2, entry 2). For comparison purposes, we also performed the reaction at 60 °C in ChCl/Gly and ChCl/urea for 3 hours which afforded product **3a** in respectively 2% and 20% yield (Table 2, entries 3 and 4). Repeating the reaction in ChCl/urea with a catalytic amount of ʟ-proline (20 mol %) only provided a slightly better yield of 30% (Table 2, entry 5). This observation demonstrates the positive role of proline on the reaction mechanism but clearly indicates that it is not sufficient. In fact, the exact role of DES in this reaction is still not clear as ʟ-proline may act as a catalyst via an iminium pathway as previously described [21–22]. On the other hand, Pro/Gly DES may also activate carbonyl groups as suggested by Mohire et al. [23] and Theresa et al*.* [24]. In any event, the DES structure in Pro/Gly seems to play an important role in promoting the reaction.

**Table 2:** Optimization of Knoevenagel conditions.^a^

entry	product	DES	conditions	yield

1	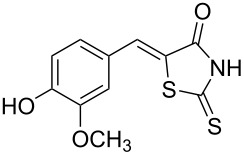 **3a**	Pro/Gly (1:2)	2 h, rt	57%
2		Pro/Gly (1:2)	1 h, 60 °C	94%
3		ChCl/Gly (1:2)	3 h, 60 °C	2%
4		ChCl/urea (1:2)	3 h, 60 °C	20%
5		ChCl/urea (1:2)^b^	3 h, 60 °C	30%
6	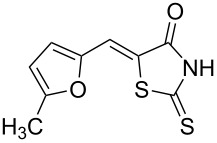 **3b**	Pro/Gly (1:2)	1 h, 60 °C	92%
7		ChCl/urea (1:2)	3 h, 60 °C	20%
8		ChCl/urea (1:2)^b^	3 h, 60 °C	75%

^a^Reactions were performed with 0.8 g of DES, 0.5 mmol of the aldehyde and 0.5 mmol of rhodanine; ^b^in the presence of 20 mol % ʟ-proline.

5-Methylfurfural was selected as another model substrate and similar results were obtained. One more time, the Knoevenagel condensation in ChCl/urea gave a poorer yield of product **3b** (20%, Table 2, entry 7) than in Pro/Gly (92%, Table 2, entry 6) unless ʟ-proline is added in catalytic amounts (Table 2, entry 8).

**Scheme 1:**
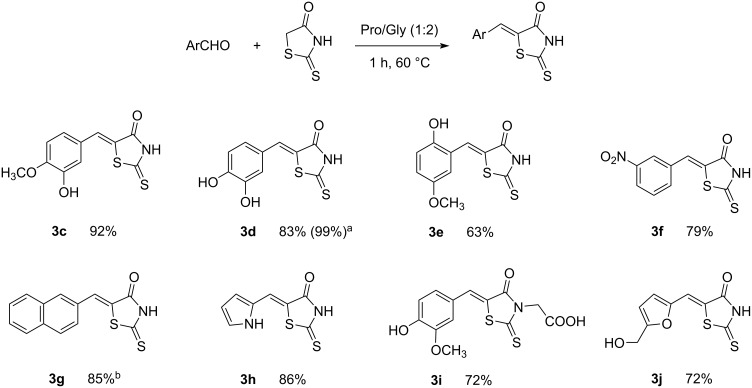
Synthesis of 5-benzylidenerhodanine derivatives. Conditions: ^a^reaction performed for 3 h at 60 °C. ^b^Reaction performed for 24 h at 60 °C.

The optimized conditions were then extended to other aldehydes to study the scope of this reaction (Scheme 1). It must be noted that in most of the cases, the reaction mixture changed its appearance from colorless to yellow or orange and that some solid precipitate formed during the reaction. The addition of water at the end of the reaction clearly led to the appearance of a solid–liquid biphasic mixture as the components of the DES are water-soluble. A large volume of water should be used since otherwise some DES traces are present in the NMR spectrum of the precipitate.

Benzaldehydes having electron-donating or electron-withdrawing groups were studied as well as heteroaromatic aldehydes. The condensation reaction with 3-hydroxy-4-methoxybenzaldehyde and rhodanine gave product **3c** with an excellent 92% yield after 1 h at 60 °C in Pro/Gly (1:2). Also, using 3,4-dihydroxybenzaldehyde gave product **3d** in 83% yield after 1 h of reaction and the product yield increased to 99% when the reaction was run for 3 h. 2-Hydroxy-5-methoxybenzaldehyde in the reaction with rhodanine gave product **3e** in a moderate 63% yield. The presence of an electron-withdrawing group such as a nitro did not seem to really decrease the reactivity as product **3f** was obtained in 79% yield. Surprisingly, 2-naphthaldehyde was found to be less reactive and needed a reaction time of 24 h to obtain product **3g** with 85% yield. Heterocyclic aldehydes gave also good results as shown with pyrrole-2-carboxaldehyde that allowed formation of product **3h** in 86% yield. Rhodanine-3-acetic acid also allowed formation of the expected 5-benzylidenerhodanine **3i** under the optimized conditions with a slightly lower yield (**3i** versus **3a**).

Finally, we also decided to investigate the reactivity of 5-hydroxymethylfurfural (HMF). Indeed, HMF is considered as one of the most promising biomass-derived platform chemicals due to its rich chemistry and readily availability from carbohydrates [25]. We synthesized HMF according to a modified procedure of Cao et al. [26]. Fructose and tetraethylammonium chloride were heated to 120 °C for 2 h and the reaction media was extracted by THF to recover crude HMF after concentration. HMF was then directly used in the Knoevenagel condensation with rhodanine in Pro/Gly yielding product **3j** in 72% yield. The overall yield for this two-step process was 36%.

As some of the products have already been described in the literature, we compared our results with the reported conditions (Table 3). As it can be seen, the yields obtained in this study are similar or better than those reported earlier. Moreover, our optimized conditions use mild temperature, short reaction times, and exclude the use of volatile organic compounds (VOC) in the workup stage. The Pro/Gly NaDES can also be recycled for several runs [20].

**Table 3:** Comparison to other reported methods for 5-benzylidenerhodanine synthesis.

product	yield obtained in this paper	method	yield	reference

**3a**	94%	PrNH_2_ (2 equiv), mw, 80 °C, 60 min ChCl/urea, 90 °C malonitrile, EtOH, Et_3_N, rt, few hours water, 90 °C, 7 d B(OH)_3_ 20 mol %, mw, 160 °C, 40 min EtOH, NH_4_OH, NH_4_Cl, 80 °C, 2 h	80% 53% 90% 52% 79% 75%	[27] [3] [5] [28] [29] [30]
**3b**	92%	AcOH, AcONa, reflux, 2 h		[31]
**3c**	92%	PrNH_2_ (2 equiv), mw, 80 °C, 60 min piperidine, EtOH, reflux, 4 h β-alanine (2 equiv), acetic acid, 100 °C, 3 h PrNH_2_ (2 equiv), mw, 60 °C, 30 min TBAB, water, mw, 10 min	81% 23% 100% 85% 77%	[27] [32] [33] [34] [35]
**3d**	83%	PrNH_2_ (2 equiv), mw, 80 °C, 60 min EtOH, piperidine, AcOH, mw, 140 °C, 30 min PrNH_2_ (2 equiv), mw, 60 °C, 30 min	79% 60% 82%	[27] [36] [34]
**3f**	79%	malonitrile, EtOH, Et_3_N, rt, few hours	94%	[5]
**3g**	85%	EtOH, AcOH, reflux, 24 h malonitrile, EtOH, Et_3_N, rt, few hours EtOH, piperidine, 70 °C, 16 h AcOH, AcONa, reflux, 24 h AcOEt, Et_3_N, AcOH, 85 °C, 3 h	84% 89% 86% 76% 50%	[4] [5] [37] [38] [39]
**3h**	86%	EtOH, AcOH, AcONa, reflux, 1 h malonitrile, EtOH, Et_3_N, rt, few hours Et_2_NH, H_2_O, rt, 5 h	72% 88% 95%	[40] [5] [41]
**3i**	72%	Amberlyst 26, ultrasound irradiation, 60 °C, 6 h	75%	[42]

As some benzylidenerhodanine derivatives were already reported for their antioxidant activities [3], we investigated those compounds for their antioxidant activity expressed as percentage of 1,1-diphenyl-2-picrylhydrazyl radical (DPPH) scavenging activity. DPPH free radicals give a purple solution and present a strong absorption maximum at 517 nm. In the presence of an antioxidant compound DPPH is reduced forming DPPH-H and the color of the solution changes to yellow. The overall antioxidant capacity of compounds was measured after 30 minutes of incubation. We used the same protocol as described by Molnar et al. [43] where DPPH and the synthesized compounds were tested in a solution at 0.2 mM concentration in DMSO as solvent.

Because phenolic compounds can be easily oxidized to quinones, it is well known that most hydroxylated compounds have antioxidant properties. Moreover, it has already been shown that a catechol-like structure greatly contributes to the antioxidant activity in DPPH scavenging activities [43]. The antioxidant activities of compounds **3a**–**h** are presented in Table 4 and our results were consistent with the observations of Molnar et al. [3], i.e., 3,4-dihydroxybenzylidenerhodanine (**3d**) was the most active derivative and substitution of the 3-OH group with a methoxy group decreased the activity (**3a** versus **3d**). Moreover, we found that substitution of the 4-OH with a methoxy group also decreased the activity (**3c** versus **3d**) to almost the same extent. Interestingly, compound **3e** with an OH group at position 2 and a methoxy substituent at position 5 was slightly more potent with 41.6% inhibition. Compounds without a OH group (like **3f**–**h**) presented as expected a weak scavenging activity.

**Table 4:** Structures and observed antioxidant activity expressed as % DPPH scavenging.^a^

compound	% inhibition DPPH	compound	% inhibition DPPH

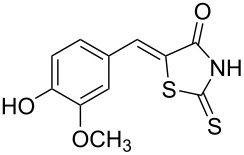 **3a**	32.3	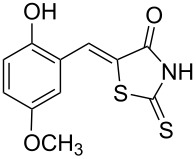 **3e**	41.6
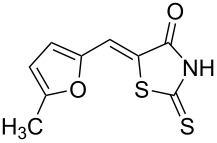 **3b**	19	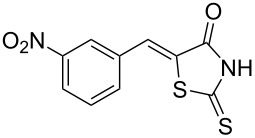 **3f**	10.7
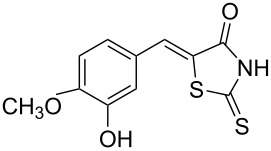 **3c**	29.2	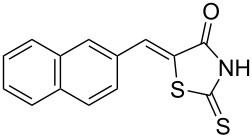 **3g**	6.8
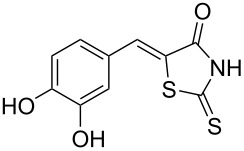 **3d**	52.7	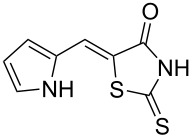 **3h**	0.9
ascorbic acid	53.4		

^a^Experiments were performed in triplicate.

## Conclusion

5-Arylidenerhodanines were successfully synthesized in an ʟ-proline-based deep eutectic solvent. Indeed, Pro/Gly (1:2) was the most effective DES compared to classical ChCl/Gly and ChCl/urea. It allowed formation of the expected products in very good yields at mild temperature (60 °C), and in most cases within only one hour of reaction. Heteroaromatic aldehydes as well as benzaldehydes with electron-donating or electron-withdrawing groups could be used as substrates. The method is fast, easy, catalyst-free, and sustainable as no classical organic solvents were used. The synthesized derivatives were studied for their antioxidant activities and as expected, all compounds with a hydroxy group showed DPPH radical scavenging activity. Compound **3d** with a catechol-like structure exhibited the best antioxidant activity.

## Experimental

### General procedure for the Knoevenagel condensation

DES (0.8 g) was introduced in a 10 mL round-bottomed flask. Then, the aldehyde (0.5 mmol) and rhodanine (0.5 mmol) were sequentially added to the DES and the reaction mixture was stirred at 60 °C for the indicated time. Then, water (10 mL) was added at room temperature and the formed precipitate was collected by filtration and washed with 10 mL of water. No further purification was needed.

**(*Z*)-5-(5-Hydroxymethylfurfurylidene)-2-thioxothiazolidin-4-one (3j)**. ochre yellow solid obtained after 1 h at 60 °C in 36% yield (two-step yield). Mp 149 °C; ^1^H NMR (400 MHz, DMSO-*d*_6_) δ (ppm) 4.49 (s, 2H), 5.52 (br s, 1H, OH), 6.58 (d, *J* = 3.6 Hz, 1H), 7.11 (d, *J* = 3.6 Hz, 1H), 7.44 (s, 1H, =CH), 13.62 (br s, 1H, NH); ^13^C NMR (100 MHz, DMSO-*d*_6_) δ (ppm) 196.8, 169.1, 161.2, 148.8, 121.8, 121.0, 117.8, 110.9, 56.0. HRMS–ESI^−^ (*m*/*z*): [M]^−^ calcd for C_9_H_6_NO_3_S_2_: 240.982814; found, 240.982805.

### DPPH-scavenging activity

Determination of antioxidant activity was performed according to the procedure described in the literature [21]. A DMSO solution of the corresponding synthesized compound (1.5 mL, 0.2 mM) was added to a DMSO solution of DPPH radicals (1.5 mL, 0.2 mM), so that the final concentration of DPPH radical and the synthesized compound in a solution was 0.1 mM. The mixture was shaken and allowed to stand at room temperature. After 30 min, the absorbance at 517 nm was determined and the scavenging activity was calculated according to Equation 1.


[1] $$scavenging\;activity\;\left(\%\right)\;=\;\left(\left(A_{b}+\;A_{s}-\;A_{m}\right)/A_{b}\right)*100$$

A_b_ – absorbance of 0.1 mM DMSO solution of DPPH radical at 517 nm; A_s_ – absorbance of 0.1 mM DMSO solution of test compound at 517 nm; A_m_ – absorbance of DMSO mixture of test compound and DPPH radical at 517 nm. Ascorbic acid (AA) was used as a reference compound.

## Supporting Information

File 1 Experimental procedures, characterization of compounds, copies of NMR spectra and HRMS spectra.
