# Supplementary material for: Synthesis of 5-arylidenerhodanines in L-proline-based deep eutectic solvent
Source: Beilstein J Org Chem. 2023 Oct 4;19:1537–44. doi: 10.3762/bjoc.19.110 (PMC10562643; doi:10.3762/bjoc.19.110)
Supplement: File 1 — Experimental procedures, characterization of compounds, copies of NMR spectra and HRMS spectra. [file Beilstein_J_Org_Chem-19-1537-s001.pdf]

BEILSTEIN JOURNAL OF ORGANIC CHEMISTRY

Supporting Information

for

Synthesis of 5-arylidenerhodanines in L-proline-based deep eutectic solvent

Stéphanie Hesse

Beilstein J. Org. Chem. **2023,** *19,* 1537–1544. doi:10.3762/bjoc.19.110

Experimental procedures, characterization of compounds, copies of NMR spectra and HRMS spectra

License and Terms: This is a supporting information file under the terms of the Creative Commons Attribution License (https://creativecommons.org/licenses/by/4.0). Please note that the reuse, redistribution and reproduction in particular requires that the author(s) and source are credited and that individual graphics may be subject to special legal provisions.
The license is subject to the *Beilstein Journal of Organic Chemistry* terms and conditions: (https://www.beilstein-journals.org/bjoc/terms)

HRMS analysis

Samples of products **3e** and **3j** were respectively dissolved in 1 mL ethanol (ethanol HPLC grade 99.9%, Carlo Erba Reagents) for a final concentration of 1 µg/mL, to be characterized by FT-ICR MS (FT-ICR MS 7T, SolariX 2xR, Bruker Daltonics, Bremen, Germany) with electrospray ionization (ESI - Bruker Daltonics), in negative-ion detection mode. Both, ion source and instrument parameters were optimized via software FTMS-Control V2.3.0 (Bruker Daltonics). Prior to acquisition, the mass spectrometer was externally calibrated and ICR detection cell was shimmed and gated using direct introduction of a 0.1 mg/mL sodium trifluoroacetate solution. The sample solution was infused into the source at a 3 µL/min flow rate and the capillary voltage was set at 3.7 kV. The drying gas temperature and flow rate were kept at 200 °C and 4 L/min, respectively, and the nebulizer pressure at 0.5 bar. The mass spectra resulted from the accumulation of 20 scans over a *m/z* 107.5–1500 range and with a 4 megaword time-domain. A mass resolution of 450 000 was achieved at *m/z* 300. Molecular formulae were assigned by SmartFormula Editor (Bruker Daltonics) with a ±1 ppm mass accuracy window. Theoretical mass specta, with the isotopic distribution, were also used to confirm the molecular assignment.

(*Z*)-5-(4-Hydroxy-3-methoxybenzylidene)-2-thioxothiazolidin-4-one (3a). Yellow solid obtained after 1 h at 60 °C in 94% yield. Mp = 219 °C; ^{1}H NMR (400 MHz, DMSO-d_6): δ (ppm) 3.84 (s, 3H, OCH_3), 6.94 (d, 1H, J = 8,4 Hz), 7.10 (dd, 1H, J = 8,4 Hz and 2 Hz), 7.16 (d, 1H, J = 2 Hz), 7.56 (s, 1H, =CH), 10.06 (br s, 1H, OH), 13.72 (br s, 1H, NH); ^{13}C NMR (100 MHz, DMSO-d_6) δ (ppm) 195.7, 169.8, 149.9, 148.1, 132.5, 125.0, 124.4, 121.4, 116.3, 114.3, 55.6.

(5-Methylfurfurylidene)-2-thioxothiazolidin-4-one (3b). Yellow solid obtained after 1 h at 60 °C in 92% yield. Mp = 210 °C; ^{1}H NMR (400 MHz, DMSO-d_6): δ (ppm) 2.41 (s, 3H, CH_3), 6.44 (m, 1H), 7.10 (d, 1H, *J* = 3.6 Hz), 7.42 (s, 1H, =CH), 13.62 (br s, 1H, NH); ^{13}C NMR (100 MHz, DMSO-d_6) δ (ppm) 196.6, 169.0, 158.5, 148.2, 121.8, 121.2, 117.8, 110.8, 13.8.

(*Z*)-5-(3-Hydroxy-4-methoxybenzylidene)-2-thioxothiazolidin-4-one (3c). Light yellow solid obtained after 1 h at 60 °C in 92% yield. Mp = 227 °C; ^{1}H NMR (400 MHz, DMSO-d_6): δ (ppm) 3.84 (s, 3H, OCH_3), 7.01 (d, 1H, *J* = 2 Hz), 7.06–7.12 (m, 2H), 7.49 (s, 1H, =CH), 9.55 (br s, 1H, OH); ^{13}C NMR (100 MHz, DMSO-d_6) δ (ppm) 195.7, 169.6, 150.5, 147.1, 132.3, 125.7, 124.4, 122.1, 116.0, 112.6, 55.7.

(*Z*)-5-(3,4-Dihydroxybenzylidene)-2-thioxothiazolidin-4-one (3d). Ochre yellow solid obtained after 1 h at 60 °C in 83% yield (3 h = 99% yield). Mp = 303 °C; ^{1}H NMR (400 MHz, DMSO-d_6): δ (ppm) 6.87 (d, 1H, *J* = 8.4 Hz), 6.98–6.99 (m, 2H), 7.46 (s, 1H, =CH), 9.54 (br s, 1H, OH), 9.96 (br s, 1H, OH), 13.69 (br s, 1H, NH); ^{13}C NMR (100 MHz, DMSO-d_6) δ (ppm) 196.2, 170.2, 149.7, 146.5, 133.2, 125.4, 124.9, 121.3, 117.1, 116.9.

(*Z*)-5-(2-Hydroxy-5-methoxybenzylidene)-2-thioxothiazolidin-4-one (3e). Orange solid obtained after 1 h at 60 °C in 63% yield. Mp = 220 °C; ^{1}H NMR (400 MHz, DMSO-d_6): δ (ppm) 3.72 (s, 3H, OCH_3), 6.76 (d, 1H, *J* = 2.8 Hz), 6.89 (d, 1H, *J* = 8.8 Hz), 6.97 (dd, 1H, *J* = 8.8 and 2.8 Hz), 7.79 (s, 1H, =CH), 10.30 (br s, 1H, OH); ^{13}C NMR (100 MHz, DMSO-d_6) δ (ppm) 196.1, 169.8, 152.5, 151.9, 127.4, 124.4, 120.2, 119.9, 117.5, 112.5, 55.7. HRMS–ESI$^-$ (*m/z*): $[M]^-$ calcd for $C_{11}H_8NO_3S_2$, 266.998464; found, 266.998482.

(*Z*)-5-(3-Nitrobenzylidene)-2-thioxothiazolidin-4-one (3f). Yellow solid obtained after 1 h at 60 °C in 79% yield. Mp = 237 °C; ^{1}H NMR (400 MHz, DMSO-d_6): δ (ppm) 7.76 (s, 1H, =CH),

7.81 (t, 1H, *J* = 8 Hz)), 7.99 (d, 1H, *J* = 7.6 Hz), 8.29 (m, 1H), 8.42 (m, 1H); ^{13}C NMR (100 MHz, DMSO-d_6) δ (ppm) 195.7, 170.2, 148.4, 135.7, 134.9, 131.0, 129.3, 128.4, 124.7, 124.5.

(*Z*)-5-(2-Naphthalen-2-ylmethylene)-2-thioxothiazolidin-4-one (3g). Yellow solid obtained after 24 h at 60 °C in 85% yield. Mp = 258 °C; ^{1}H NMR (400 MHz, DMSO-d_6): δ (ppm) 7.60–7.64 (m, 3H), 7.77 (s, 1H), 7.99 (d, 1H, *J* = 1.2 and 7.6 Hz), 8.04–8.08 (m, 2H), 8.19 (s, 1H);^{13}C NMR (100 MHz, DMSO-d_6) δ (ppm) 196.1, 170.0, 133.4, 132.9, 131.6, 131.5, 130.7, 129.2, 128.9, 128.3, 127.8, 127.3, 126.3, 126.2.

(*Z*)-5-(1*H*-Pyrrol-2-ylmethylene)-2-thioxothiazolidin-4-one (3h). Orange solid obtained after 1 h at 60 °C in 86% yield. Mp = 259 °C; ^{1}H NMR (400 MHz, DMSO-d_6): δ (ppm) 6.40 (m, 1H), 6.53 (m, 1H), 7.27 (d, 1H, *J* = 1.2 Hz), 7.50 (s, 1H, =CH), 11.80 (br s, 1H, NH), 13.54 (br s, 1H, NH); ^{13}C NMR (100 MHz, DMSO-d_6) δ (ppm) 194.9, 169.4, 127.3, 125.8, 121.9, 117.1, 115.0, 112.8.

(*Z*)-5-(4-Hydroxy-3-methoxybenzylidene)-4-oxo-2-thioxo-1,3-thiazolidin-3-yl)acetic acid (3i). Yellow solid obtained after 1 h at 60 °C in 72% yield. Mp = 249 °C; ^{1}H NMR (400 MHz, DMSO-d_6): δ (ppm) 3.85 (s, 3H, OCH_3), 4.67 (s, 2H, NCH_2), 6.97 (d, 1H, *J* = 8.4 Hz), 7.17 (dd, 1H, *J* = 8.4 Hz and 2 Hz), 7.23 (d, 1H, *J* = 2 Hz), 7.79 (s, 1H, =CH), 10.23 (br s, 1H, OH); ^{13}C NMR (100 MHz, DMSO-d_6) δ (ppm) 193.1, 167.5, 166.6, 150.5, 148.2, 134.7, 125.5, 124.3, 117.5, 116.5, 114.7, 55.7, 45.5.

(*Z*)-5-(5-Hydroxymethylfurfurylidene)-2-thioxothiazolidin-4-one (3j). Ochre yellow solid obtained after 1 h at 60 °C in 72% yield. Mp = 149 °C; ^{1}H NMR (400 MHz, DMSO-d_6): δ (ppm) 4.49 (s, 2H), 5.52 (br s, 1H, OH), 6.58 (d, 1H, *J* = 3.6 Hz), 7.11 (d, 1H, *J* = 3.6 Hz), 7.44 (s, 1H, =CH), 13.62 (br s, 1H, NH); ^{13}C NMR (100 MHz, DMSO-d_6) δ (ppm) 196.8, 169.1, 161.2, 148.8, 121.8, 121.0, 117.8, 110.9, 56.0. HRMS–ESI$^-$ (*m*/*z*): [M]$^-$ calcd for $C_9H_6NO_3S_2$, 240.982814; found, 240.982805.

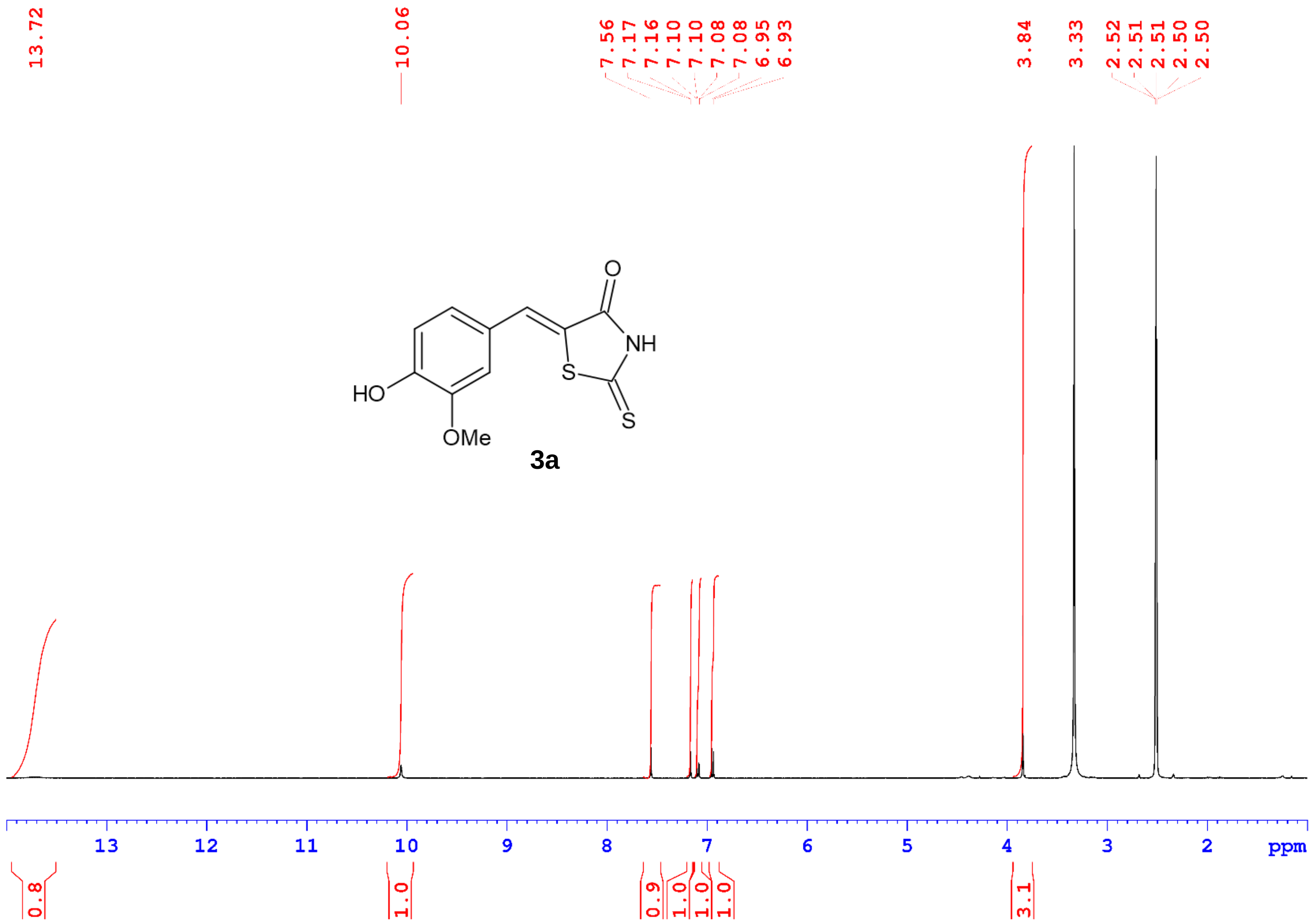
13.72
10.06
7.56
7.17
7.16
7.10
7.10
7.08
7.08
6.95
6.93
3.84
3.33
2.52
2.51
2.51
2.50
2.50
O
NH
HO
S
S
OMe
3a
13
12
11
10
9
8
7
6
5
4
3
2
ppm
0.8
1.0
0.9
1.0
1.0
1.0
3.1

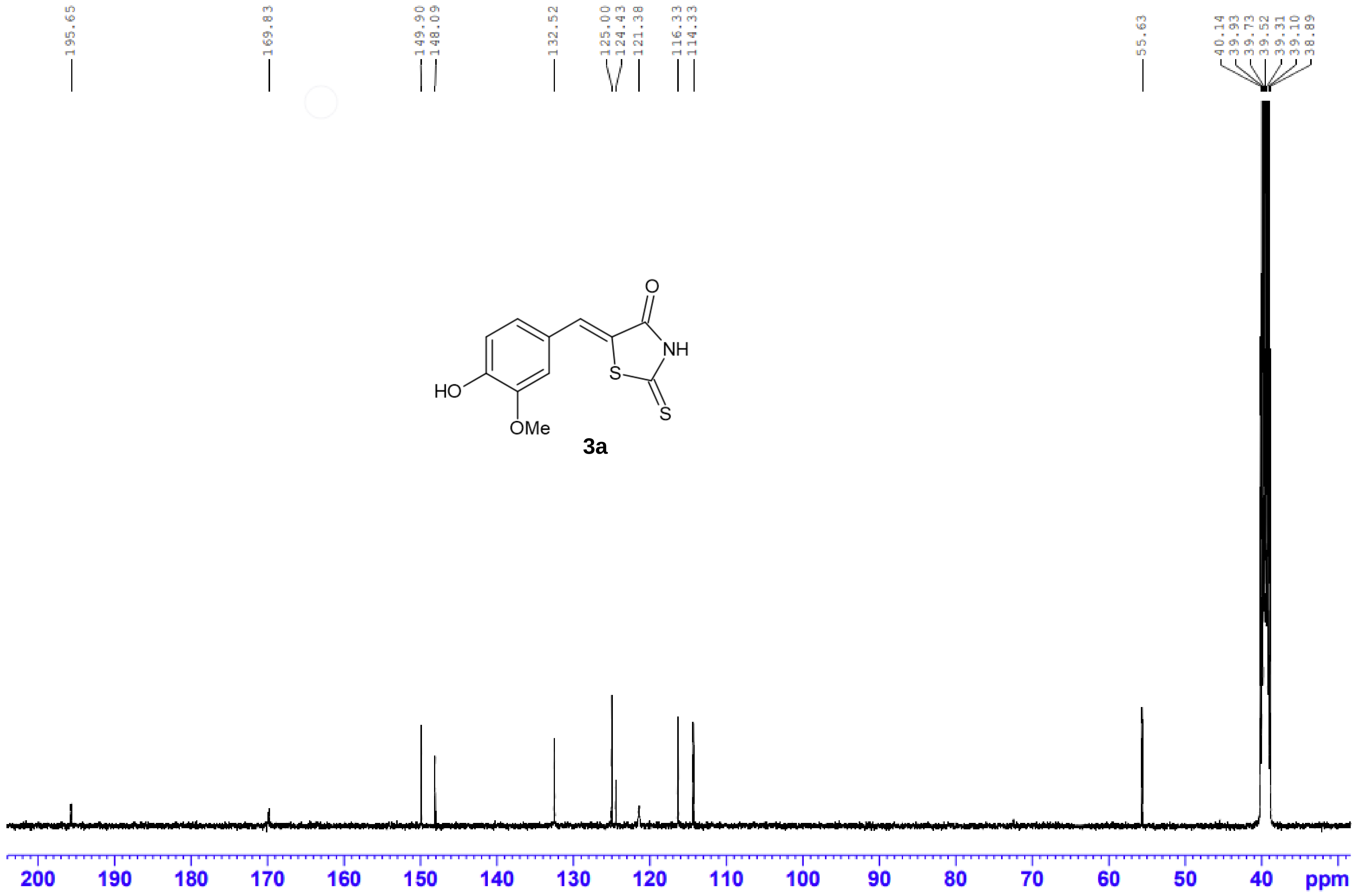
195.65
169.83
149.90
148.09
132.52
125.00
124.43
121.38
116.33
114.33
55.63
40.14
39.93
39.73
39.52
39.31
39.10
38.89
O
NH
S
S
HO
OMe
3a
200
190
180
170
160
150
140
130
120
110
100
90
80
70
60
50
40
ppm

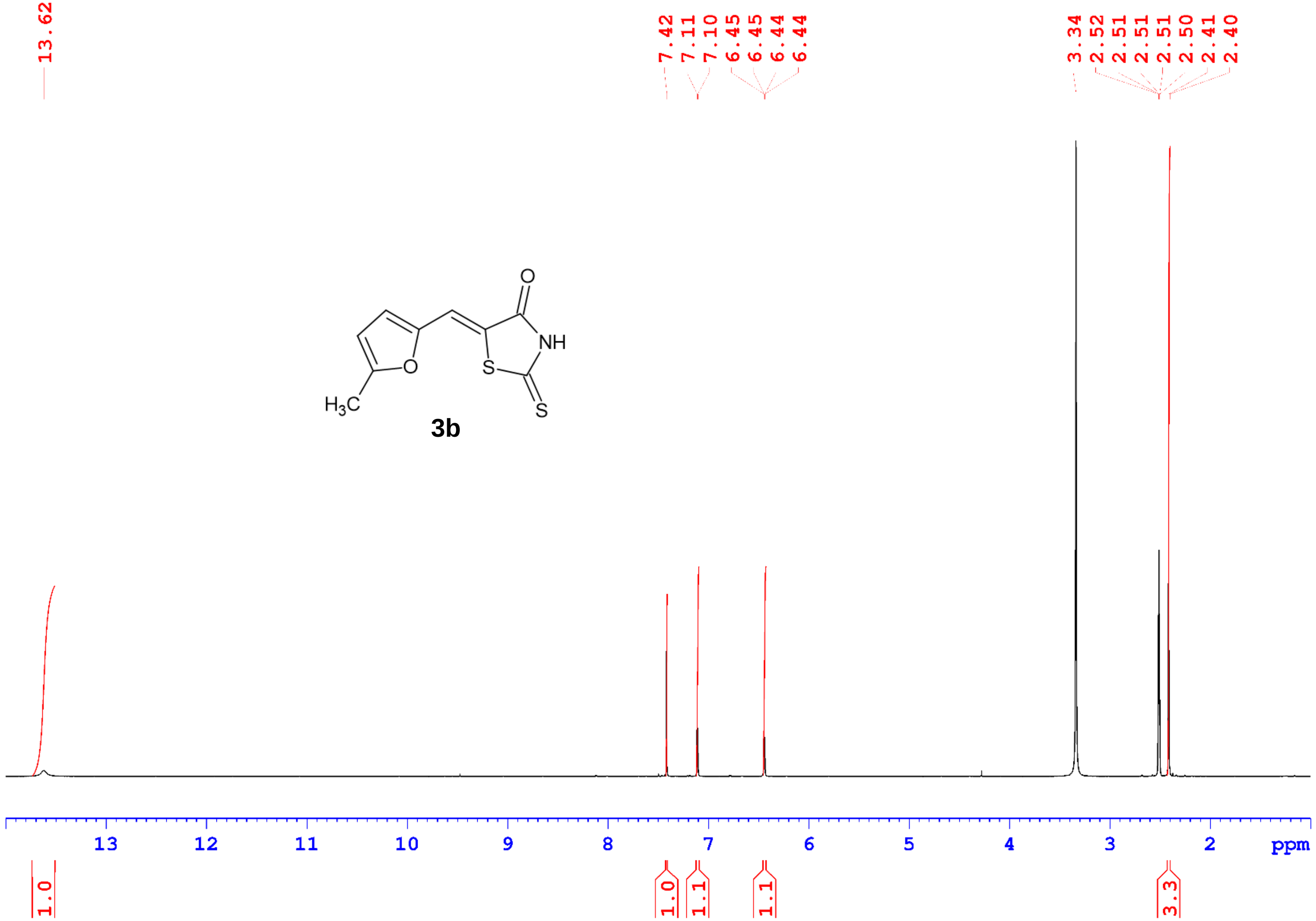
13.62
7.42
7.11
7.10
6.45
6.45
6.44
6.44
3.34
2.52
2.51
2.51
2.51
2.50
2.41
2.40
O
NH
S
O
S
H3C
3b
13
12
11
10
9
8
7
6
5
4
3
2
ppm
1.0
1.0
1.1
1.1
3.3

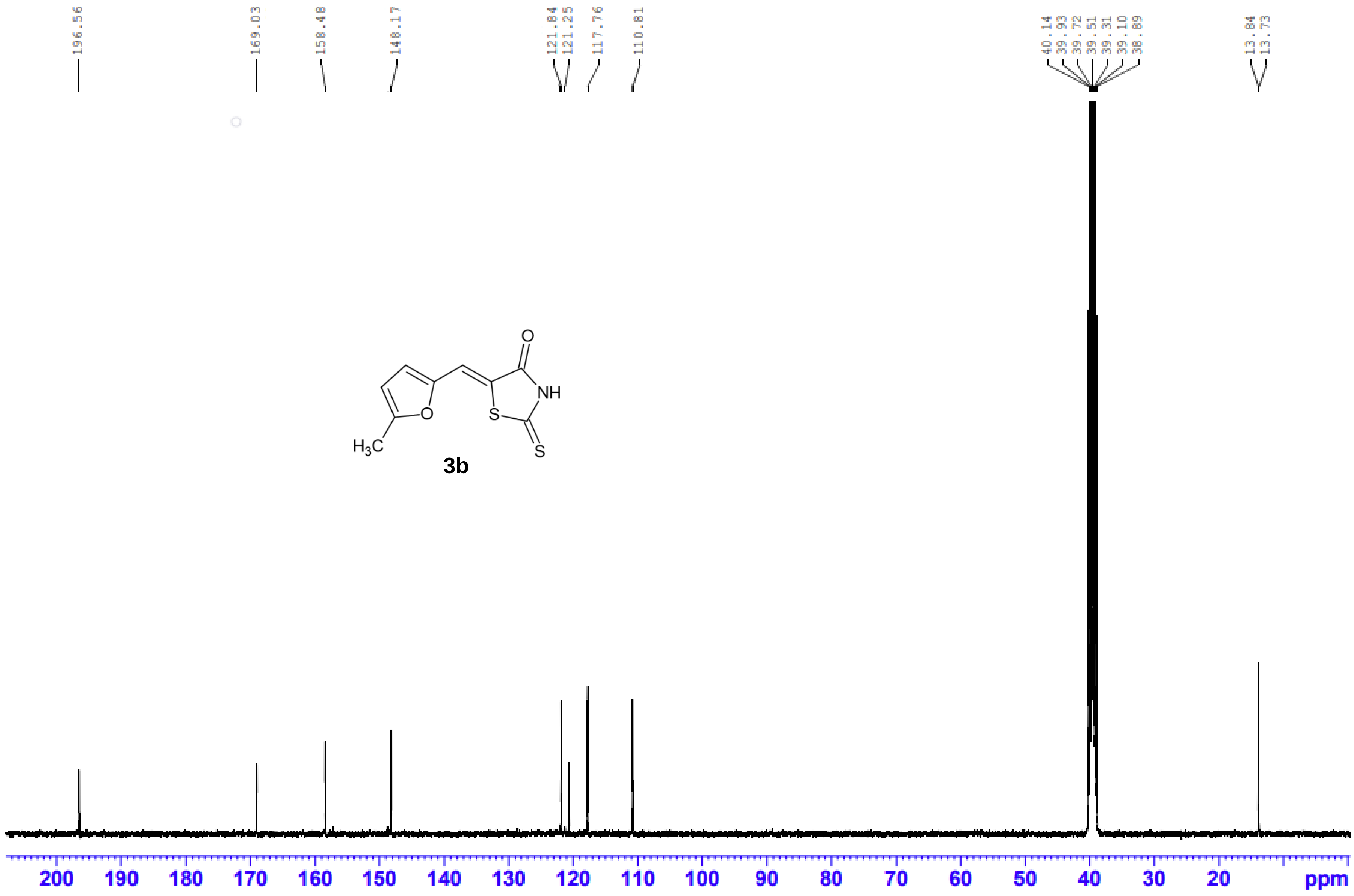
196.56
169.03
158.48
148.17
121.84
121.25
117.76
110.81
40.14
39.93
39.72
39.51
39.31
39.10
38.89
13.84
13.73
O
NH
S
S
O
H3C
3b
200 190 180 170 160 150 140 130 120 110 100 90 80 70 60 50 40 30 20 ppm

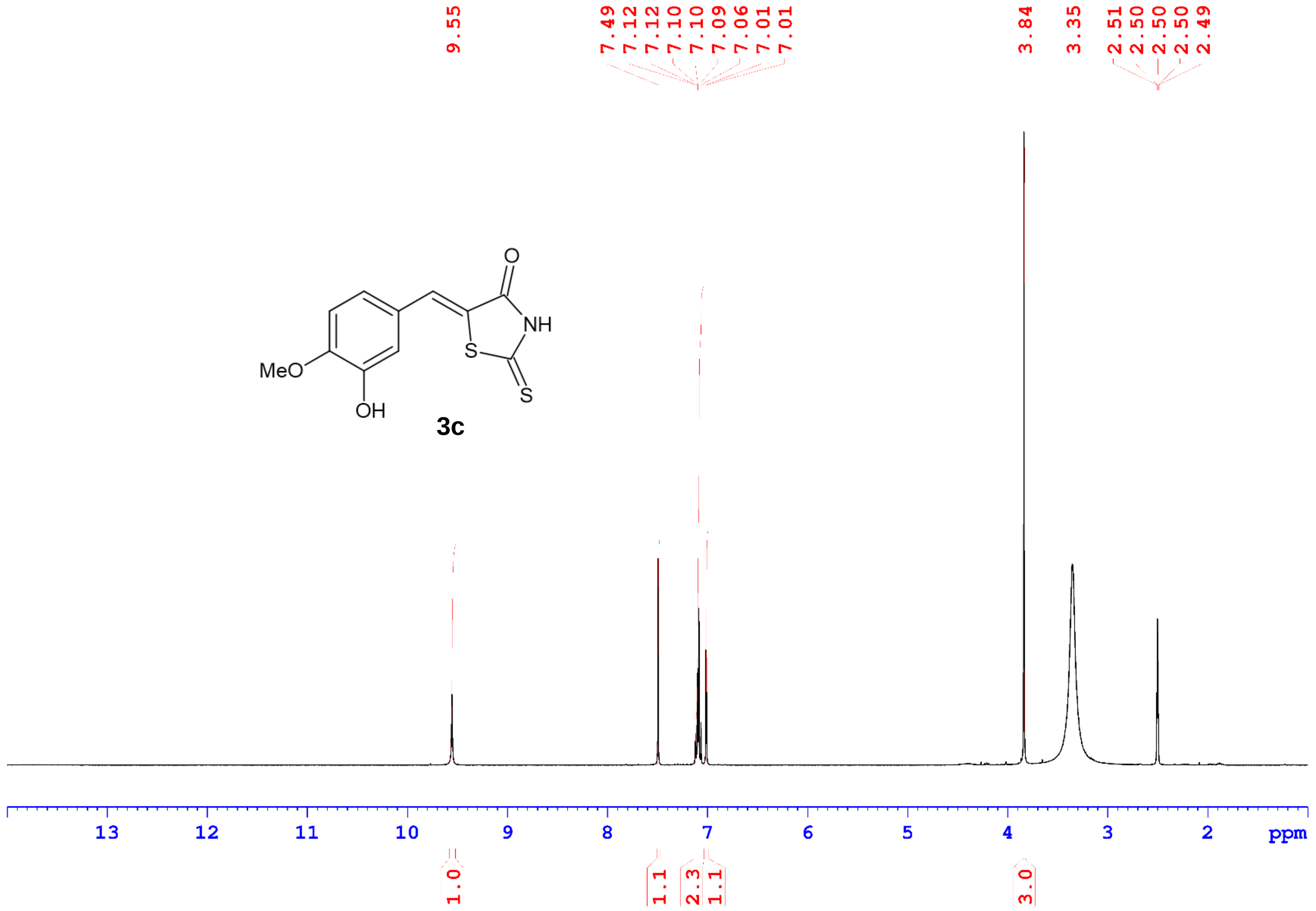
9.55
7.49
7.12
7.12
7.10
7.10
7.09
7.06
7.01
7.01
3.84
3.35
2.51
2.50
2.50
2.50
2.49
O
NH
S
S
MeO
OH
3c
13
12
11
10
9
8
7
6
5
4
3
2
ppm
1.0
1.1
2.3
1.1
3.0

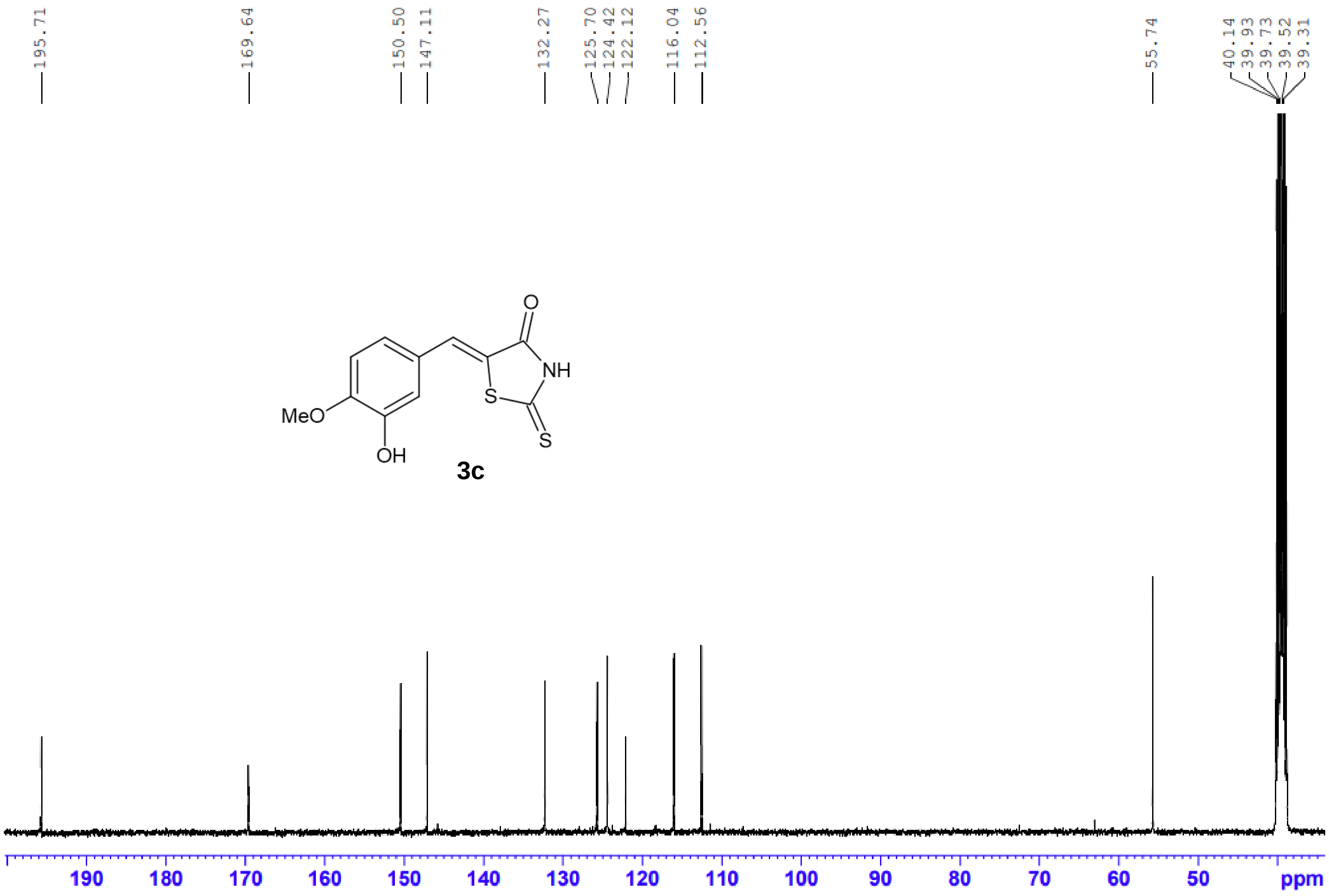
195.71
169.64
150.50
147.11
132.27
125.70
124.42
122.12
116.04
112.56
55.74
40.14
39.93
39.73
39.52
39.31
O
NH
S
S
MeO
OH
3c
190
180
170
160
150
140
130
120
110
100
90
80
70
60
50
ppm

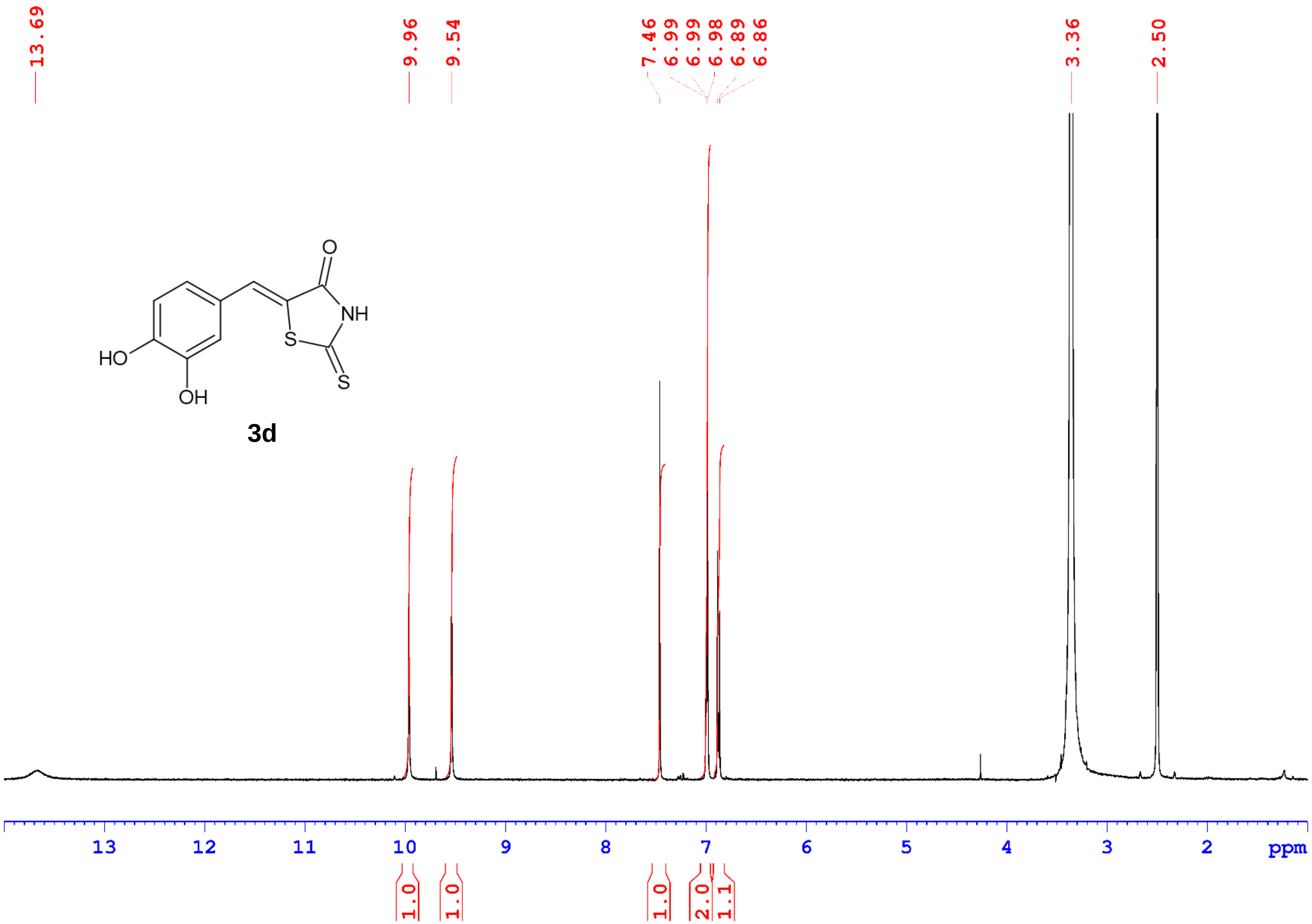
13.69
9.96
9.54
7.46
6.99
6.99
6.98
6.89
6.86
3.36
2.50
HO
OH
O
NH
S
S
3d
13
12
11
10
9
8
7
6
5
4
3
2
ppm
1.0
1.0
1.0
2.0
1.1

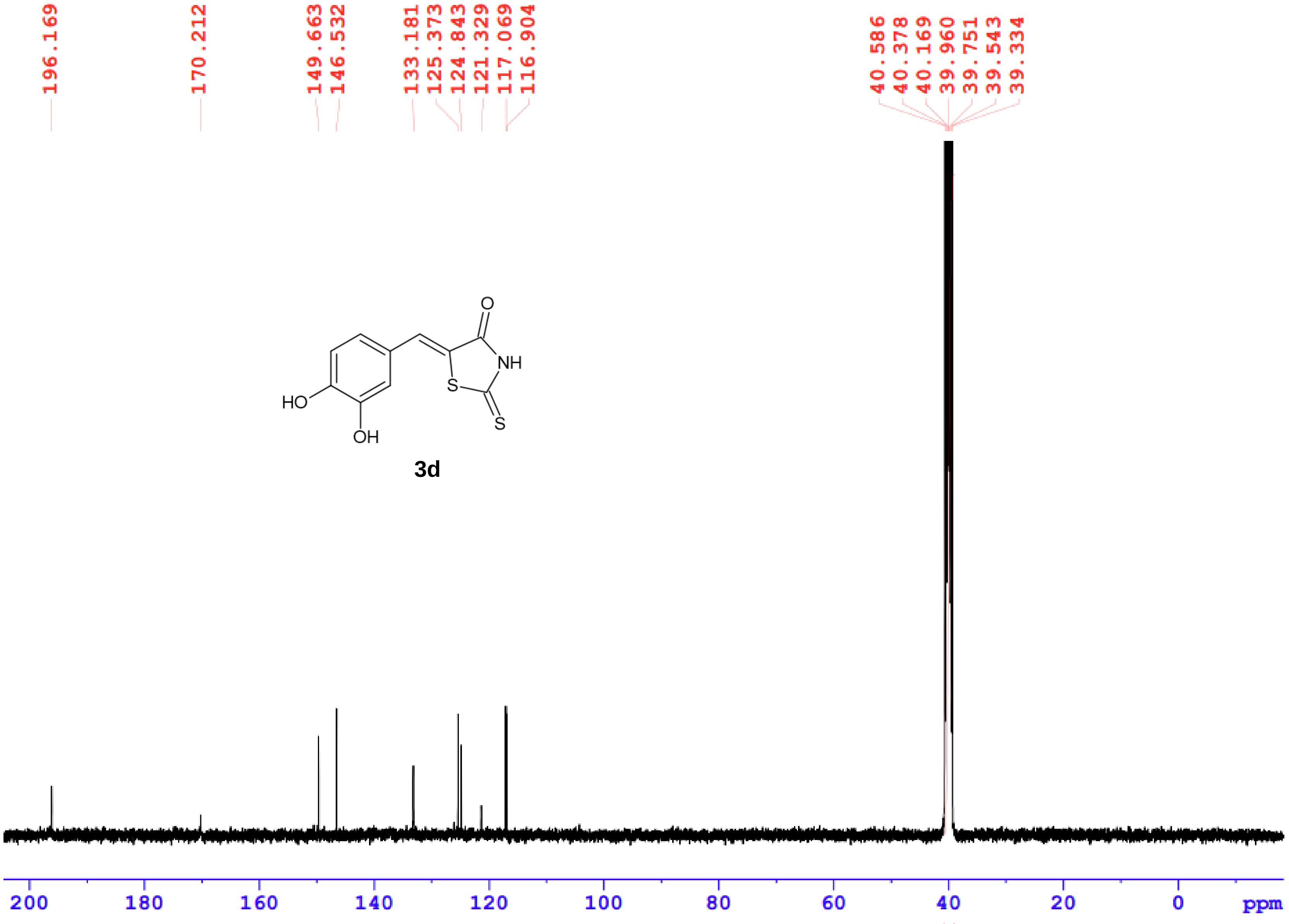
196.169
170.212
149.663
146.532
133.181
125.373
124.843
121.329
117.069
116.904
40.586
40.378
40.169
39.960
39.751
39.543
39.334
HO
OH
O
NH
S
S
3d
200
180
160
140
120
100
80
60
40
20
0
ppm

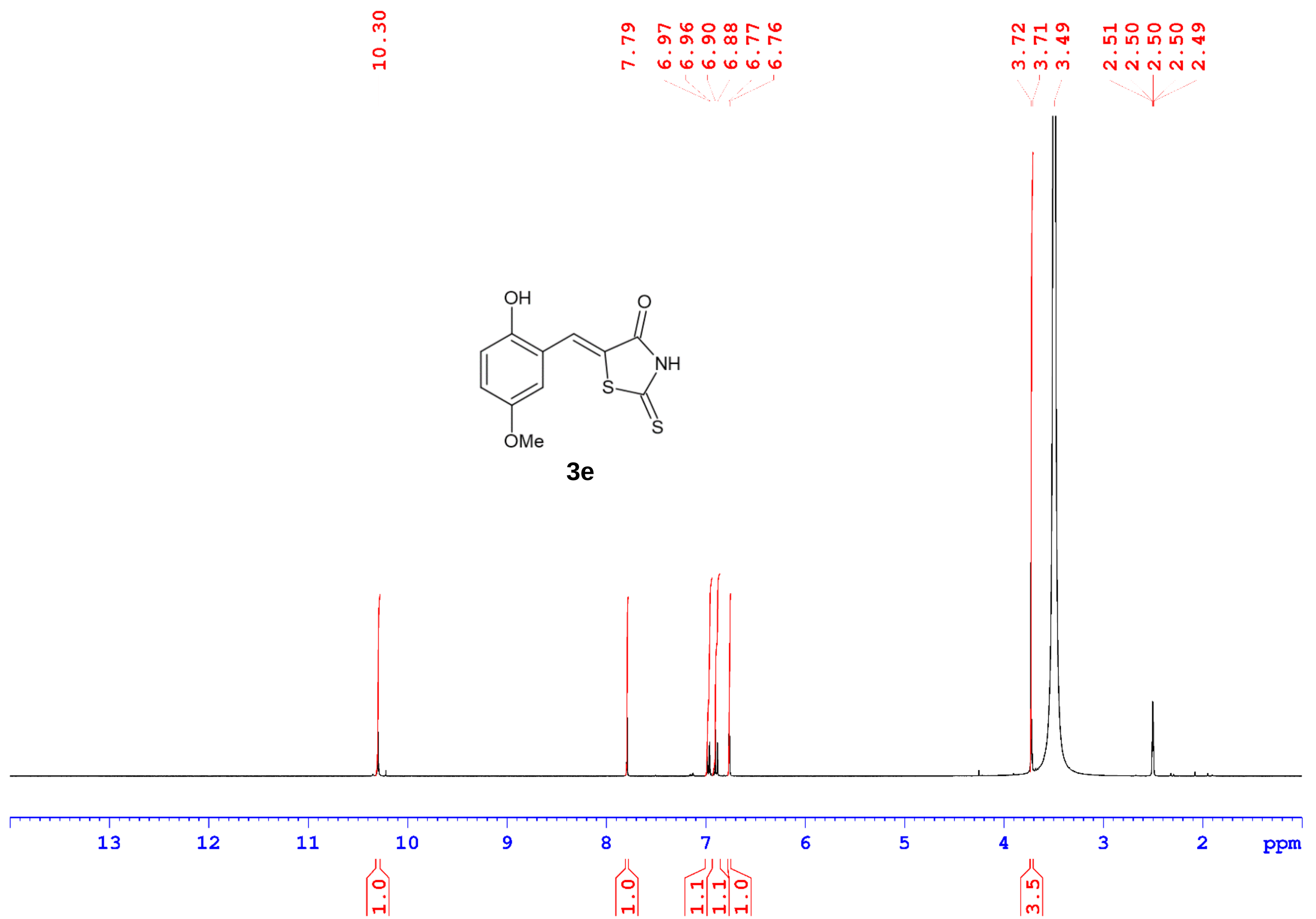

10.30
7.79
6.97
6.96
6.90
6.88
6.77
6.76
3.72
3.71
3.49
2.51
2.50
2.50
2.50
2.49
OH
O
NH
S
S
OMe
3e
13
12
11
10
9
8
7
6
5
4
3
2
ppm
1.0
1.0
1.1
1.1
1.0
3.5

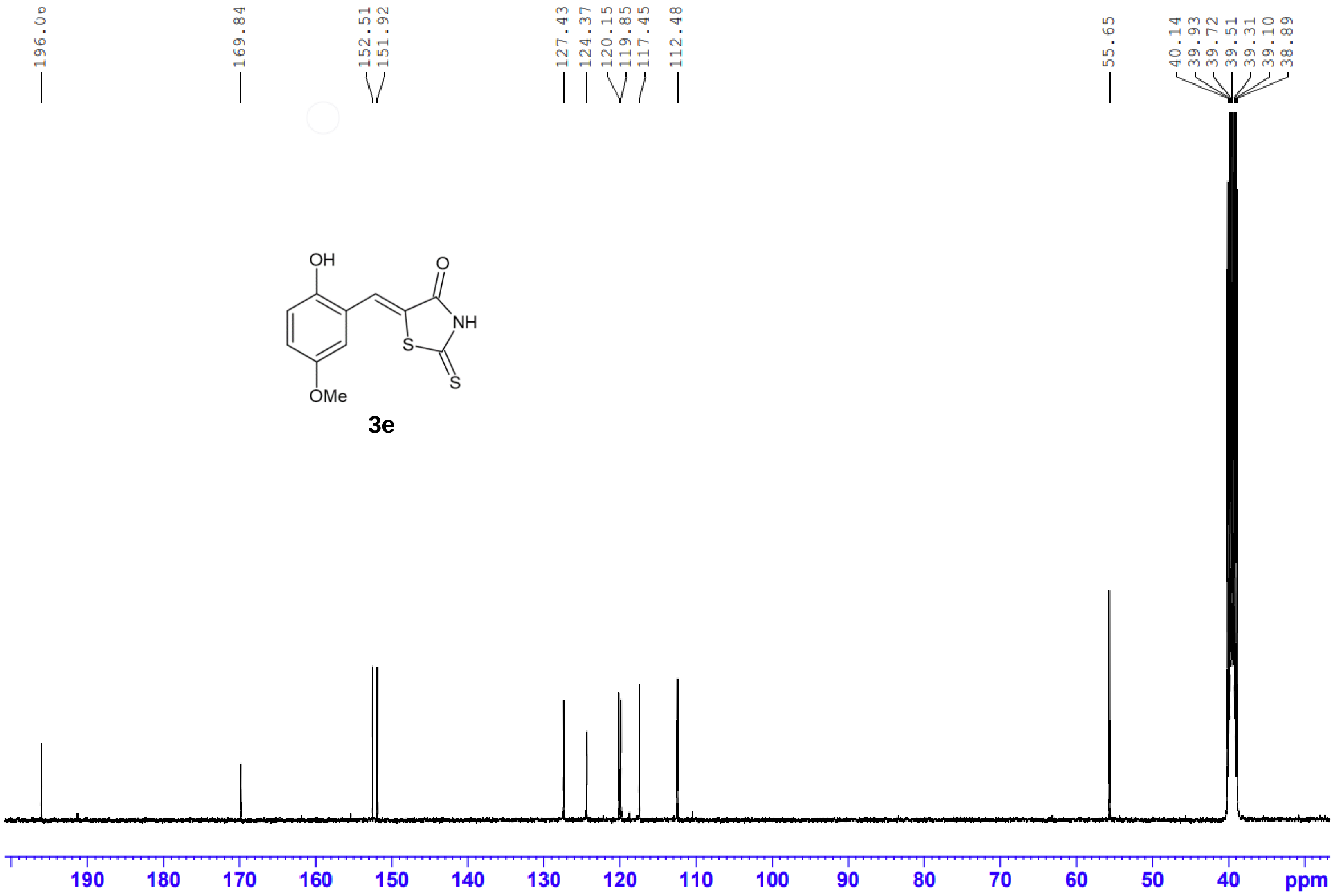
196.06
169.84
152.51
151.92
127.43
124.37
120.15
119.85
117.45
112.48
55.65
40.14
39.93
39.72
39.51
39.31
39.10
38.89
OH
O
NH
S
S
OMe
3e
190
180
170
160
150
140
130
120
110
100
90
80
70
60
50
40
ppm

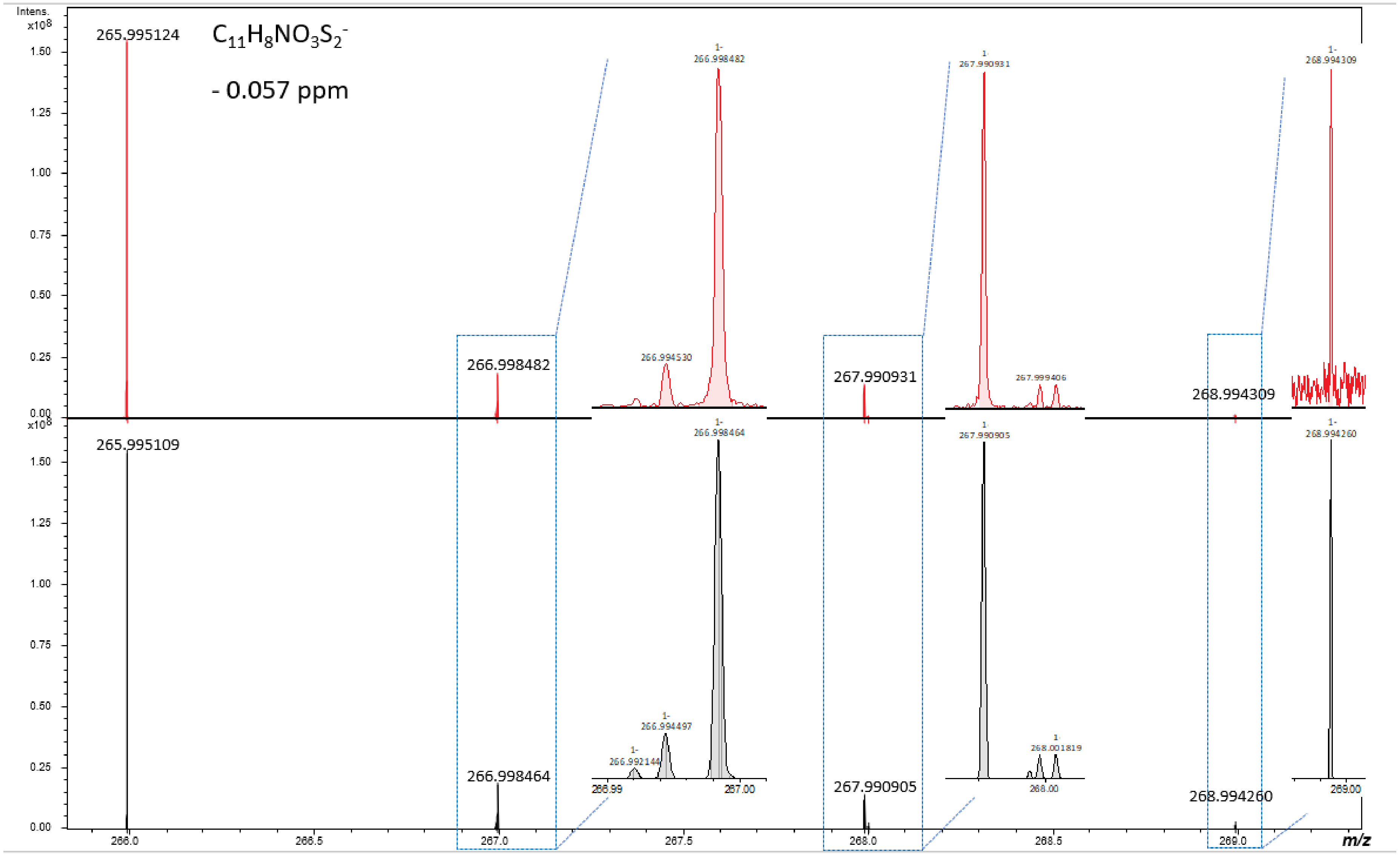

Experimental (red) and theoretical (black) isotopic distributions for 3e [$C_{11}H_8NO_3S_2$]$^-$ ion detected by ESI (-) FT-ICR MS.

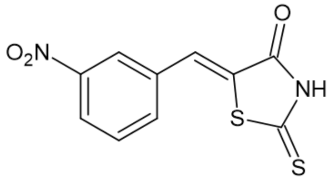
O2N
O
NH
S
S

3f

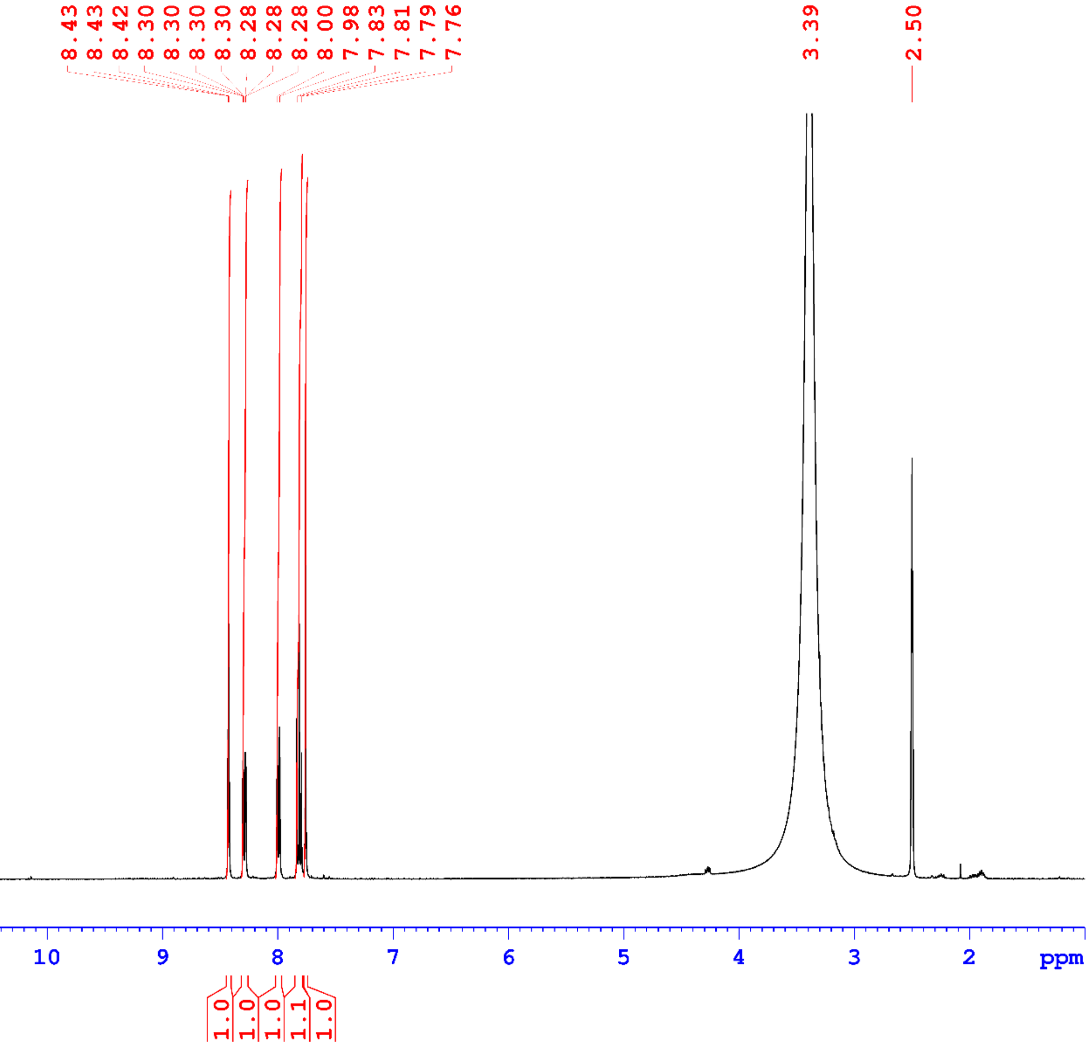
8.43
8.43
8.42
8.30
8.30
8.30
8.30
8.28
8.28
8.28
8.00
7.98
7.83
7.81
7.79
7.76
3.39
2.50
13
12
11
10
9
8
7
6
5
4
3
2
ppm
1.0
1.0
1.0
1.1
1.0

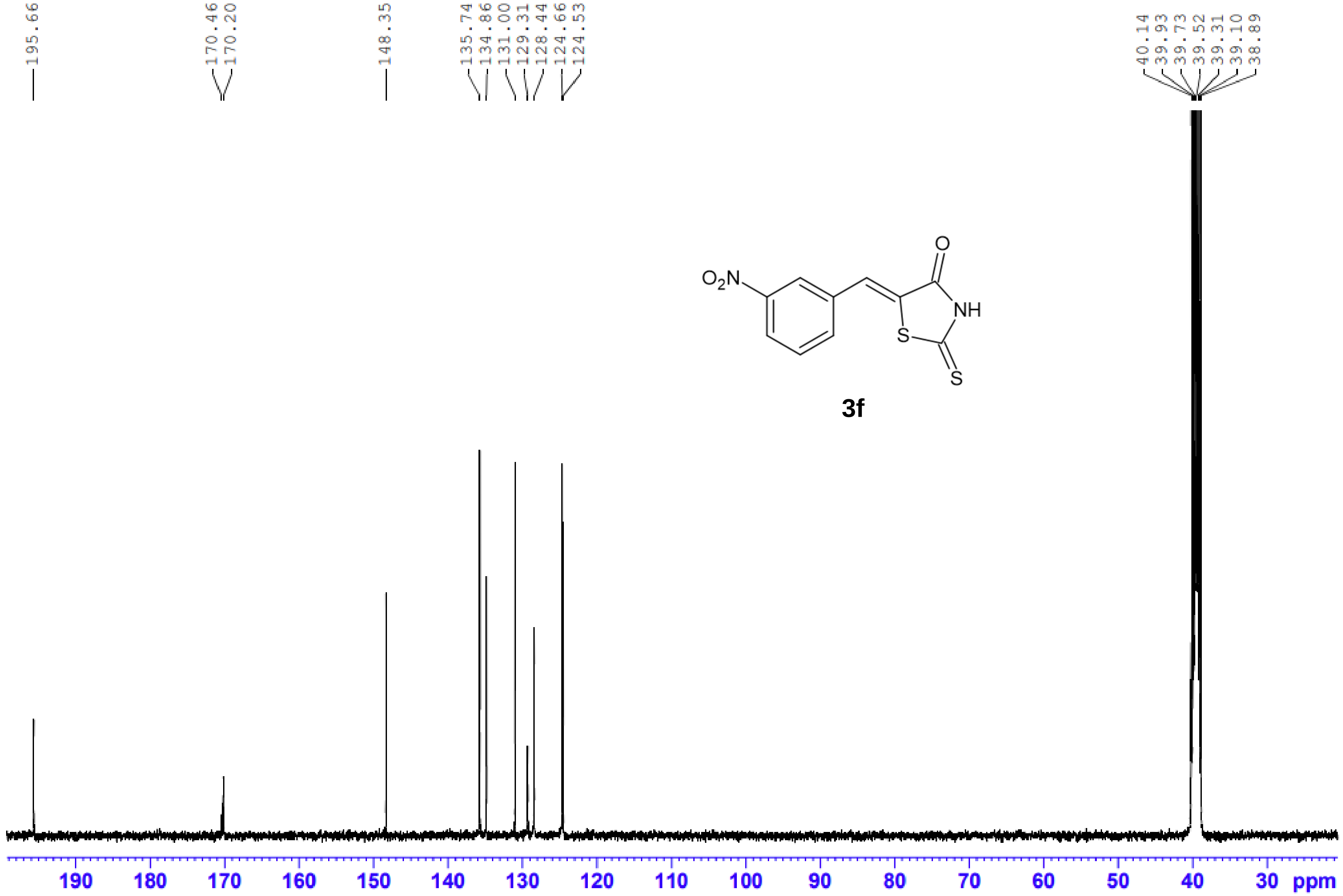
195.66
170.46
170.20
148.35
135.74
134.86
131.00
129.31
128.44
124.66
124.53
40.14
39.93
39.73
39.52
39.31
39.10
38.89
O$_2$N
O
NH
S
S
3f
190 180 170 160 150 140 130 120 110 100 90 80 70 60 50 40 30 ppm

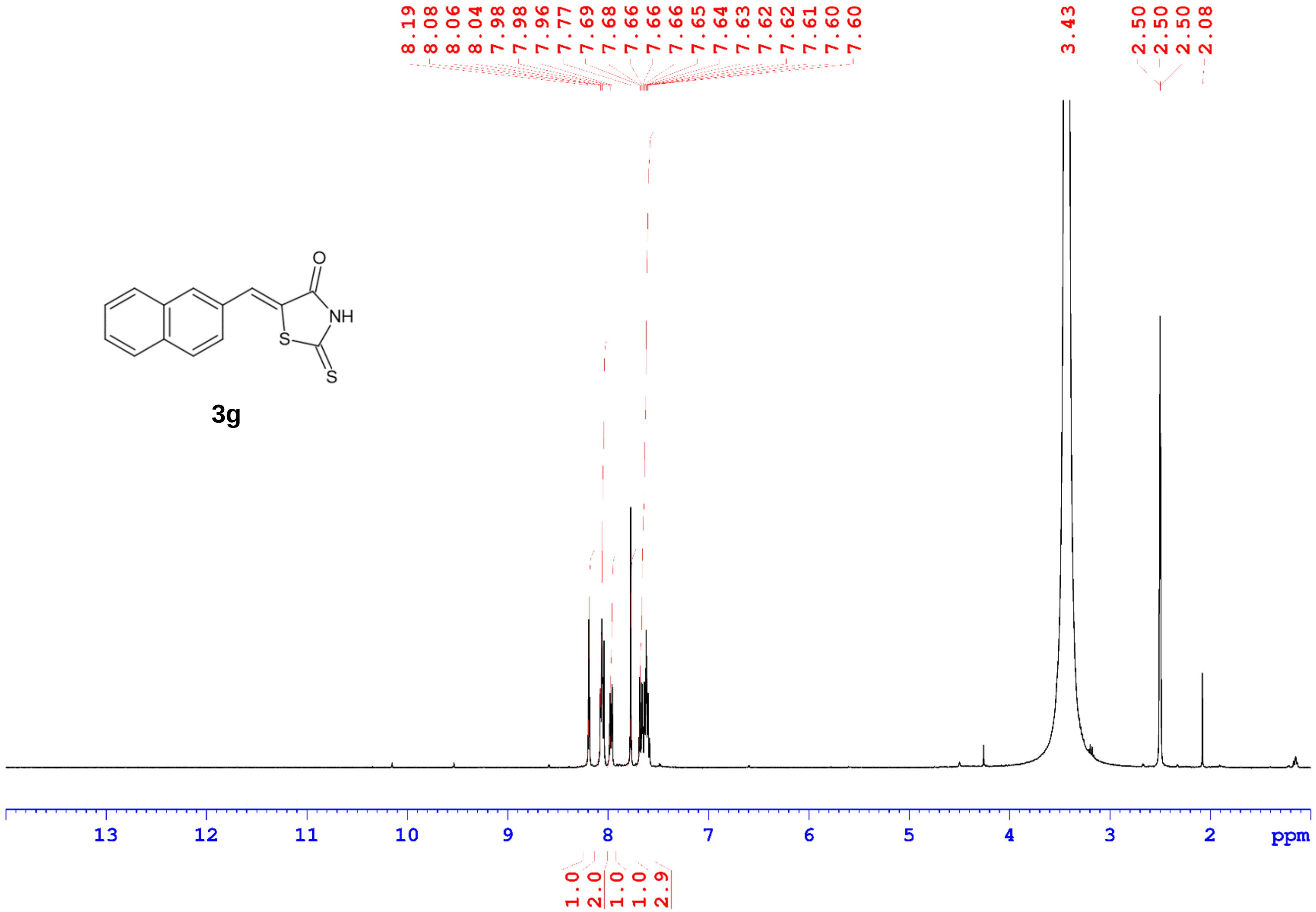

8.19 8.08 8.06 8.04 7.98 7.98 7.96 7.77 7.69 7.68 7.66 7.66 7.66 7.65 7.64 7.63 7.62 7.62 7.61 7.60 7.60
3.43
2.50 2.50 2.50 2.08
13 12 11 10 9 8 7 6 5 4 3 2 ppm
1.0 2.0 1.0 1.0 2.9

3g

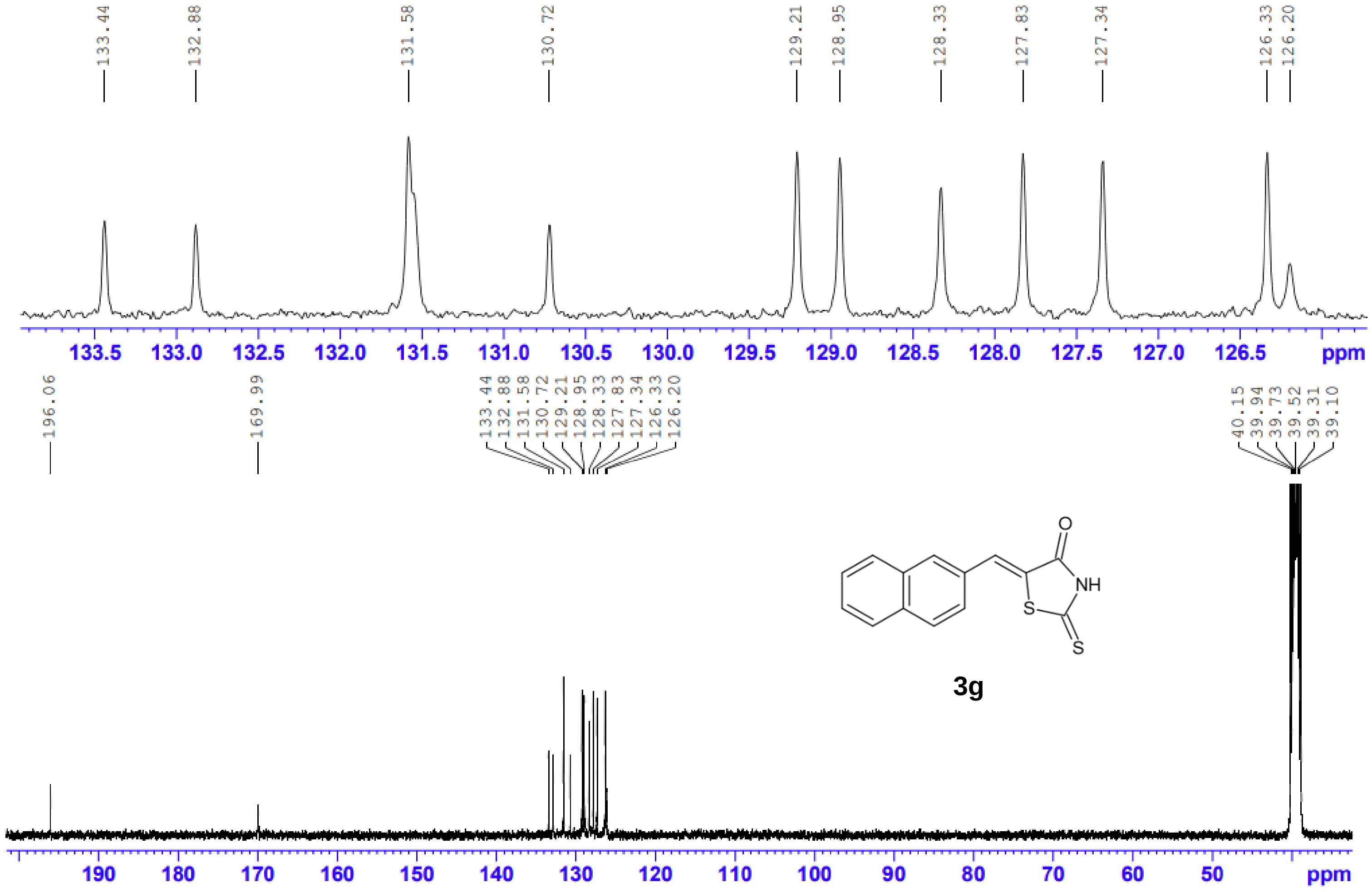
133.44
132.88
131.58
130.72
129.21
128.95
128.33
127.83
127.34
126.33
126.20
133.5 133.0 132.5 132.0 131.5 131.0 130.5 130.0 129.5 129.0 128.5 128.0 127.5 127.0 126.5 ppm
196.06
169.99
133.44
132.88
131.58
130.72
129.21
128.95
128.33
127.83
127.34
126.33
126.20
40.15
39.94
39.73
39.52
39.31
39.10
O
NH
S
S
3g
190 180 170 160 150 140 130 120 110 100 90 80 70 60 50 ppm

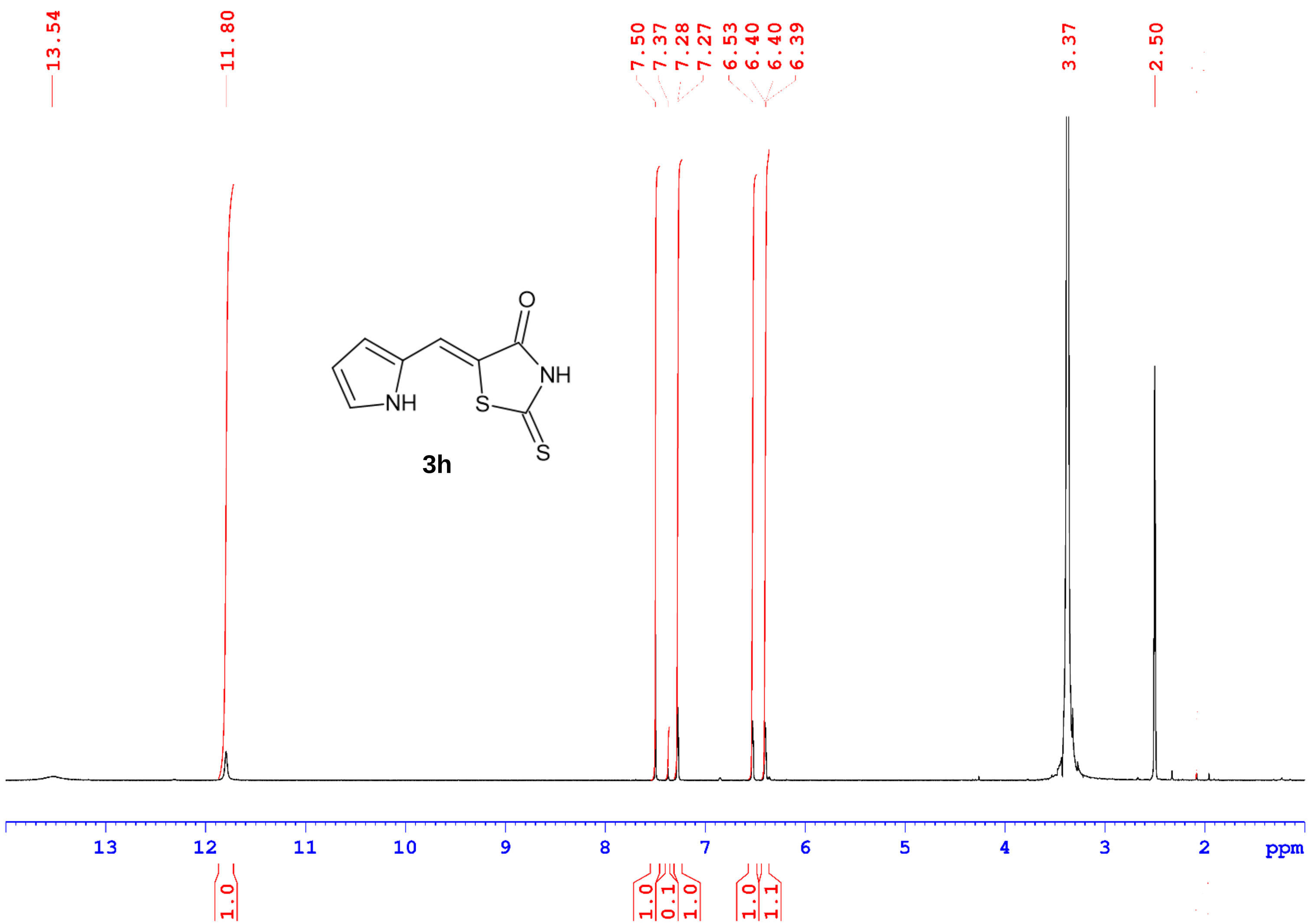
13.54
11.80
7.50
7.37
7.28
7.27
6.53
6.40
6.40
6.39
3.37
2.50
O
NH
NH
S
S
3h
13
12
11
10
9
8
7
6
5
4
3
2
ppm
1.0
1.0
0.1
1.0
1.0
1.1

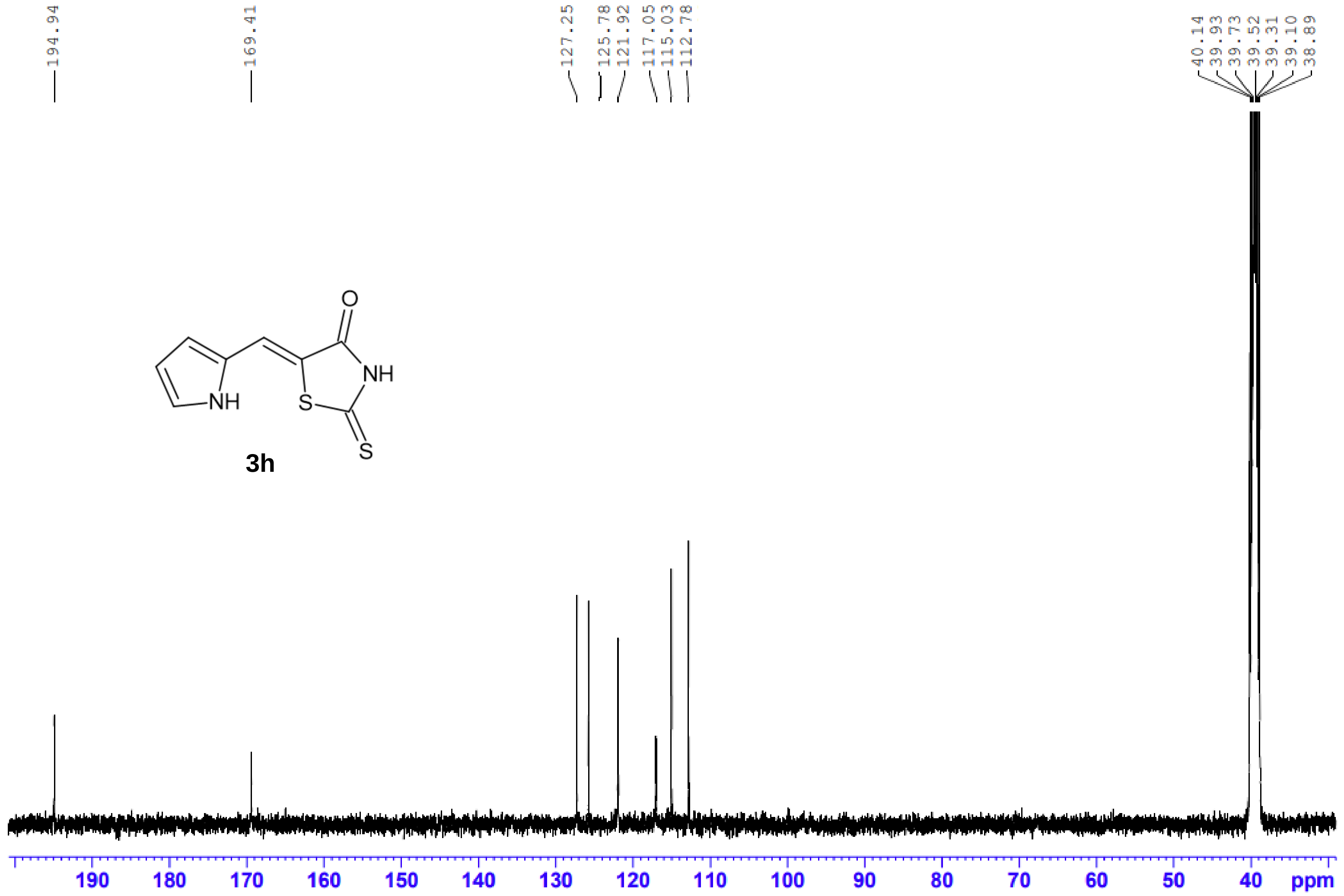
194.94
169.41
127.25
125.78
121.92
117.05
115.03
112.78
40.14
39.93
39.73
39.52
39.31
39.10
38.89
O
NH
S
NH
S
3h
190
180
170
160
150
140
130
120
110
100
90
80
70
60
50
40
ppm

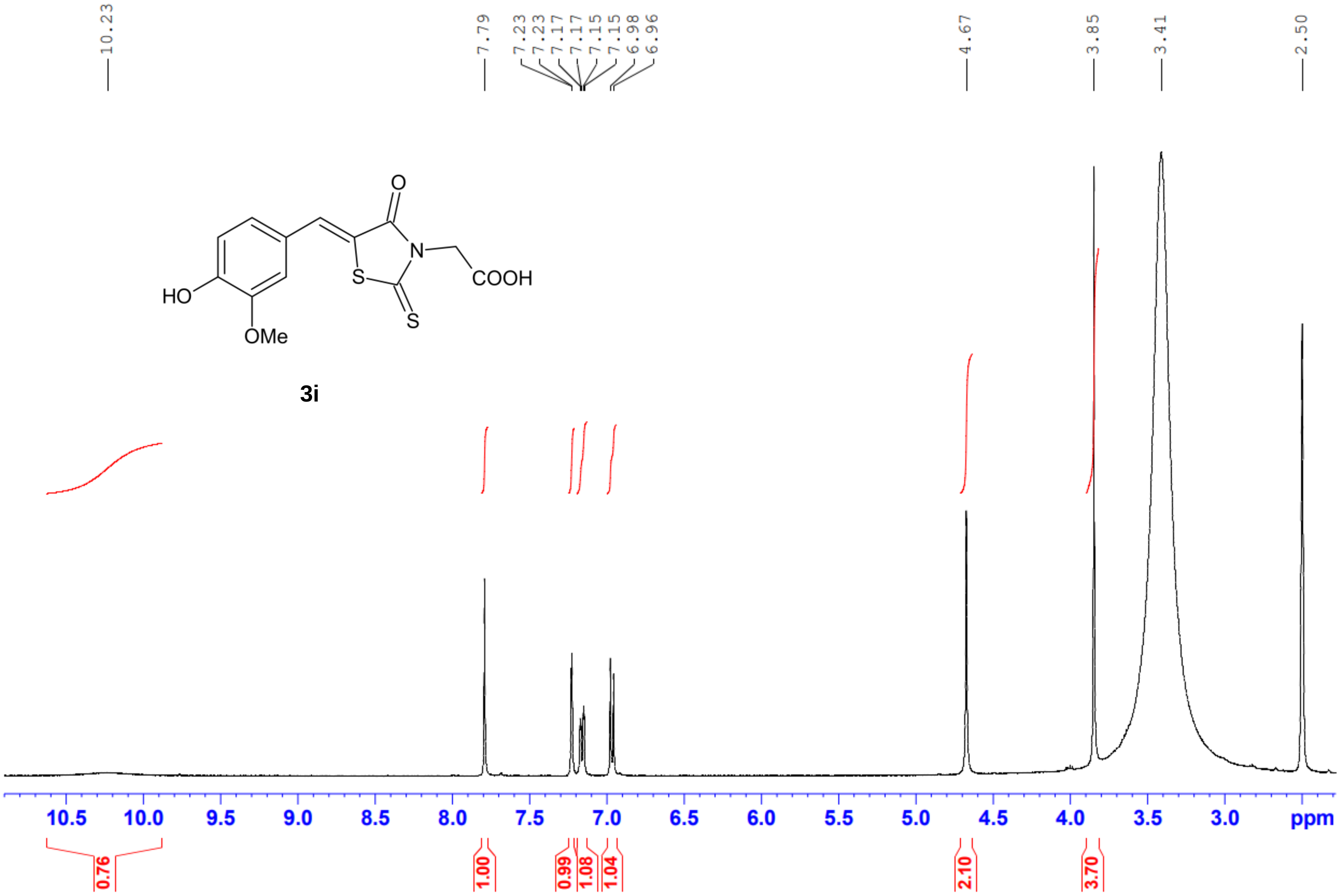
10.23
7.79
7.23
7.23
7.17
7.17
7.15
7.15
6.98
6.96
4.67
3.85
3.41
2.50
O
N
COOH
S
S
HO
OMe
3i
10.5
10.0
9.5
9.0
8.5
8.0
7.5
7.0
6.5
6.0
5.5
5.0
4.5
4.0
3.5
3.0
ppm
0.76
1.00
0.99
1.08
1.04
2.10
3.70

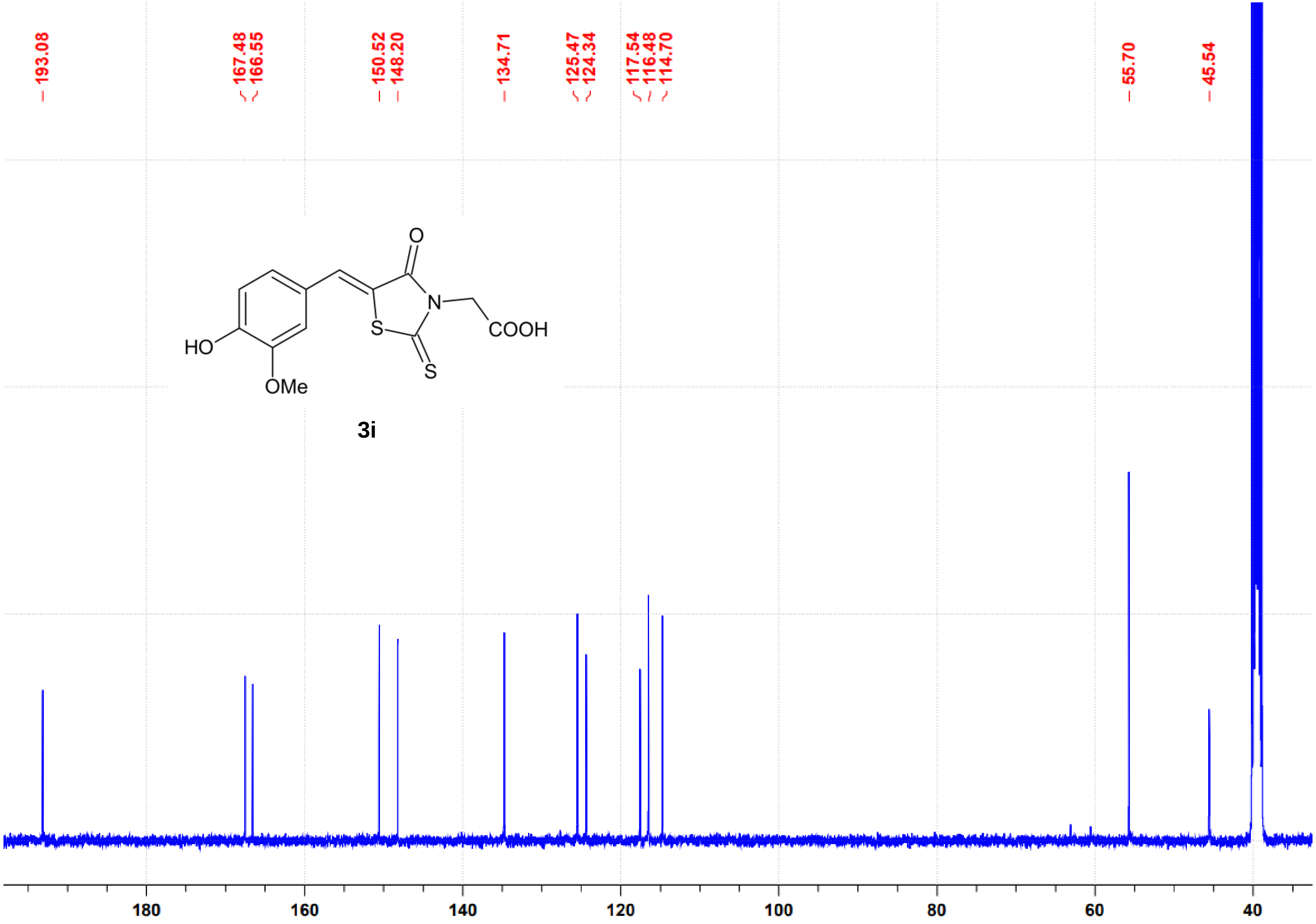
193.08
167.48
166.55
150.52
148.20
134.71
125.47
124.34
117.54
116.48
114.70
55.70
45.54
O
N
COOH
HO
S
S
OMe
3i
180
160
140
120
100
80
60
40

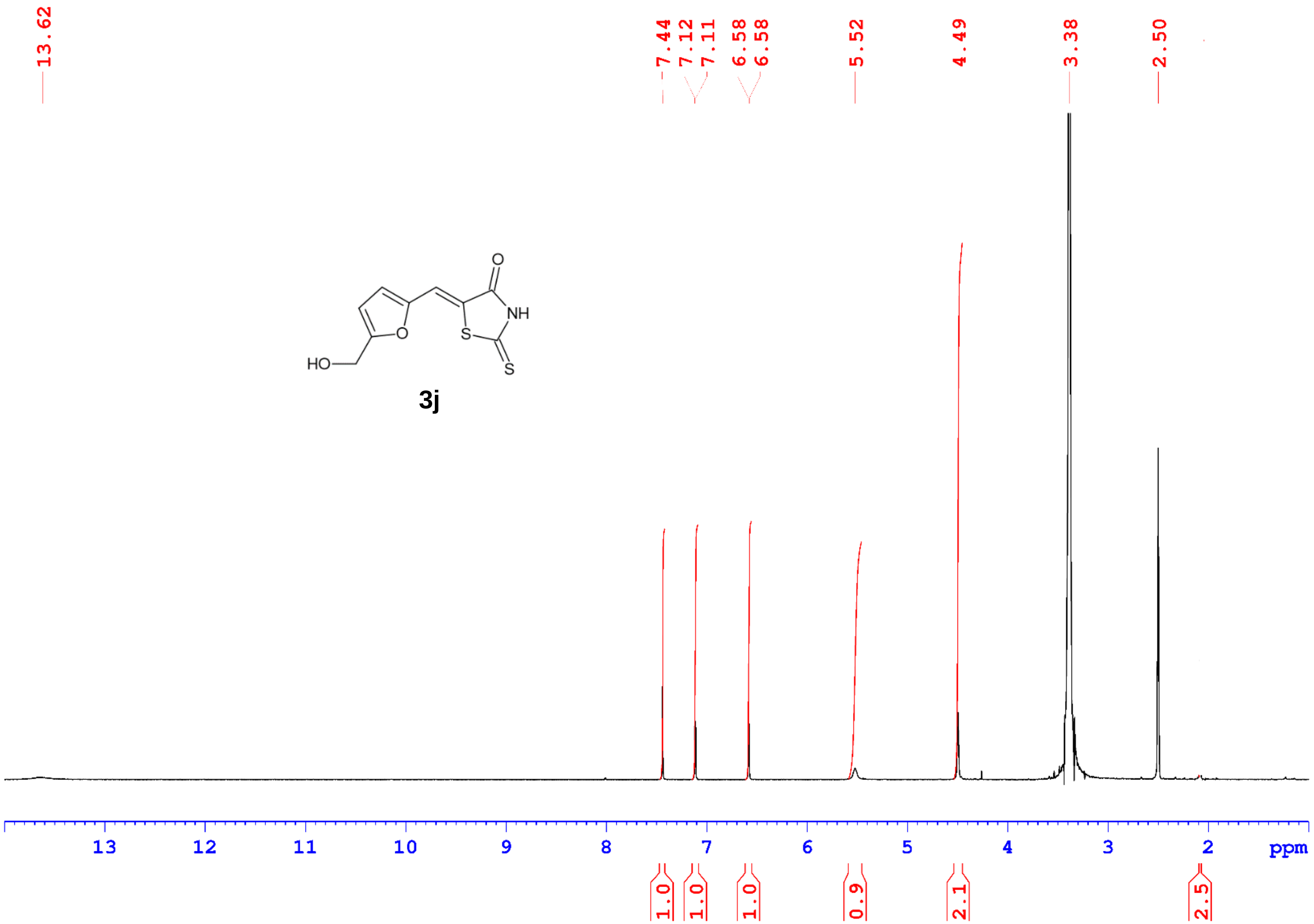
13.62
7.44
7.12
7.11
6.58
6.58
5.52
4.49
3.38
2.50
O
NH
S
S
O
HO
13
12
11
10
9
8
7
6
5
4
3
2
ppm
1.0
1.0
1.0
0.9
2.1
2.5

3j

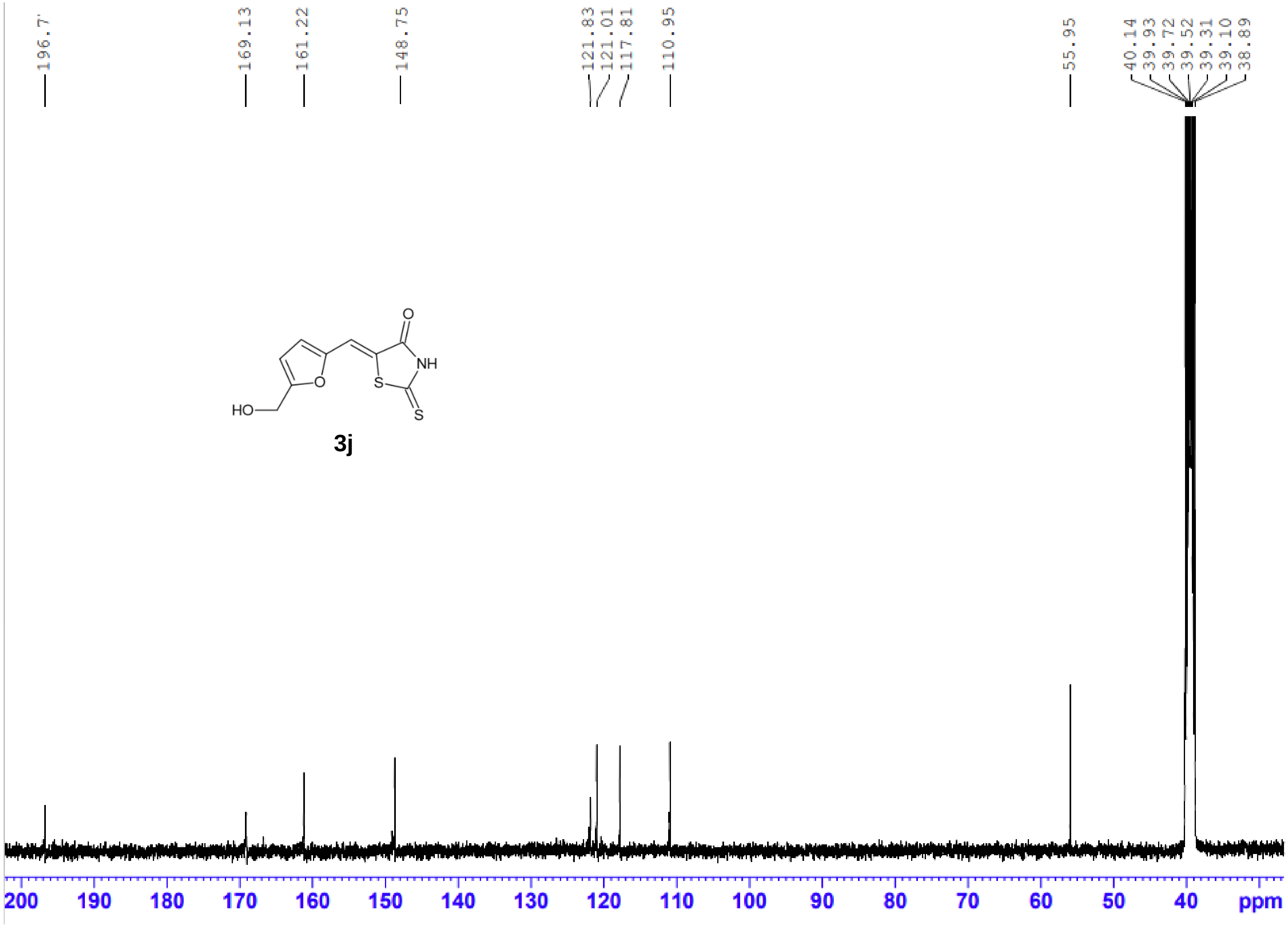
196.7
169.13
161.22
148.75
121.83
121.01
117.81
110.95
55.95
40.14
39.93
39.72
39.52
39.31
39.10
38.89
O
NH
S
S
O
HO
3j
200 190 180 170 160 150 140 130 120 110 100 90 80 70 60 50 40 ppm

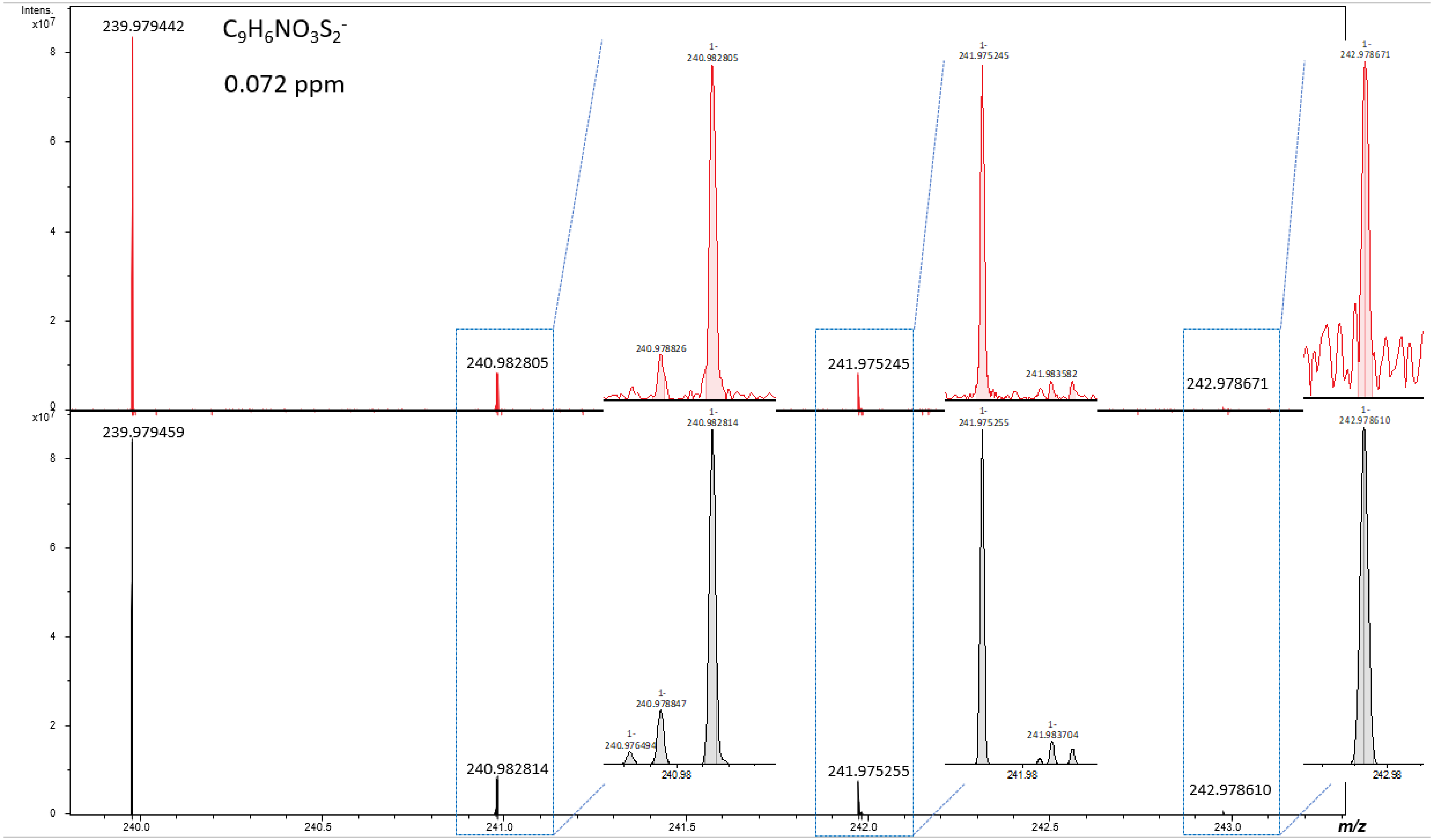

Experimental (red) and theoretical (black) isotopic distributions for **3j** $[C_9H_6NO_3S_2]^-$ ion detected by ESI(−) FT-ICR MS.
